# Novel approach for development and optimization of microgreens-based functional dairy beverage: Antioxidant enhancement, consumer acceptability, and kinetic shelf-life modeling

**DOI:** 10.1016/j.fochx.2025.102559

**Published:** 2025-05-22

**Authors:** Mahendra Gunjal, Atul Khalangre, Jyoti Singh, Sezai Ercisli, Prasad Rasane

**Affiliations:** a Department of Food Technology and Nutrition, School of Agriculture, Lovely Professional University, Phagwara, Punjab, India; b Interdepartmental Centre for Agri-Food Industrial Research, Alma mater Studiorum, University of Bologna, Via Q. Bucci 336, 47522 Cesena, (FC), Italy; c Department of Horticulture, Erzurum, Ataturk University, Turkey

**Keywords:** Antioxidant, Dairy beverage, Degradation kinetics, Functional foods, Microgreens, Shelf life, Temperature

## Abstract

This study optimizes a microgreens-based dairy (lassi) beverage using response surface methodology to enhance antioxidant composition, sensory attributes, and shelf stability. The ideal formulation (9.189 % microgreen juice, 60 % dahi, and 15.311 % water) achieved a desirability score of 0.781, significantly improving bioactive compounds. The optimized lassi contained phenolics (44.14 mg GAE/100 g), flavonoids (22.62 mg QUE/100 g), anthocyanins (37.91 μmol/100 g), and ascorbic acid (59.32 mg/100 g), with an acceptability rating of 8.29. Kinetic modeling assessed bioactive stability under varying storage conditions, with the highest retention at 5 °C after 15-days. Temperature-dependent degradation rates were analyzed using the Arrhenius, Eyring, and Ball models, while shelf-life prediction was based on a zero-order reaction model combined with the Arrhenius equation. The estimated shelf life at 5 °C based on microbial load was 9.33 days (t_1/2_), with production cost, aligning with market trends. These findings highlight commercial potential of radish sango microgreens in developing a nutritious, antioxidant-rich lassi beverage.

## Highlights

• Development of microgreens-based dairy (lassi) beverage was performed using response surface methodology.
• The optimized product showed high desirability.
• Optimized beverage had better antioxidant properties.
• Analyzed the stability using kinetic modeling of beverage
• The production costs of optimized lassi corresponds with market trends.

## Graphical abstract

**Figure:**
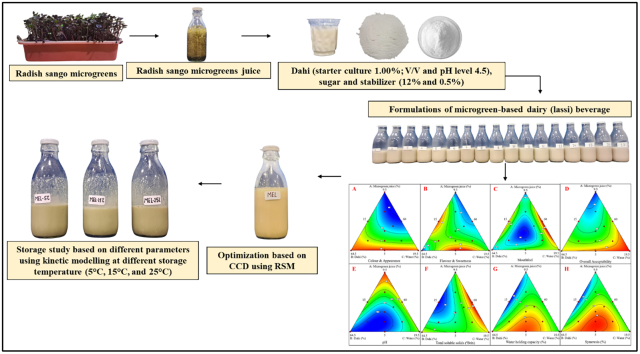

## 1 Introduction

The rising interest in functional foods and beverages is driven by increasing consumer awareness of the relationship between diet and health. Dairy products, especially fermented beverages such as Mishti Doi, Shrikhand, Buttermilk, and Lassi, are highly valued for their probiotic benefits and potential for fortification with bioactive compounds (Hussain, Patil, Yadav, & Singh, 2014). Traditional dairy products are often enhanced with functional ingredients to improve nutritional, sensory, and functional properties (Rasane, Kailey, & Singh, 2017). Fermentation significantly enhances nutrient bioavailability by promoting the activity of lactic acid bacteria, which can remove toxic and anti-nutritional compounds (Kumar, Meena, Rai, & Duary, 2023).

Microgreens, recognized for their high bioactive content, provide greater nutritional benefits than their mature counterparts. These young vegetable and herb seedlings are rich in phenolics, flavonoids, vitamins, and antioxidants, making them ideal for incorporation into functional dairy beverages (Singh et al., 2024). Recent studies have explored microgreens in various food applications, including beverages (Sharma et al., 2020; Sharma et al., 2021), seasonings, probiotic-infused products, yogurt, and bakery items (Gunjal et al., 2024b). Integrating microgreens into lassi enhances its nutritional profile while catering to the increasing demand for plant-based functional foods (Raje-Nimbalkar, Patil, Raskar, Kadav, & Joshi, 2023). However, optimizing microgreens-based dairy (lassi) requires balancing sensory attributes, nutritional quality, and storage stability.

Fermentation plays a key role in improving the functional properties of dairy products (Zhi et al., 2017). The metabolic activity of lactic acid bacteria, including *Lactobacillus*, *Streptococcus*, *Enterococcus*, and *Lactococcus*, along with probiotic strains such as *Bifidobacterium* and yeast species like *Saccharomyces* spp., enhances nutrient bioavailability and stabilizes bioactive compounds (Shah, Mokashe, & Mishra, 2016). Additionally, fermentation contributes to desirable sensory characteristics, such as texture and flavor, which are crucial for consumer acceptance (Ścibisz, Ziarno, & Mitek, 2019). The incorporation of radish sango microgreens into the fermentation process presents challenges, including maintaining bioactive compound stability and minimizing potential off-flavors (Singh et al., 2024).

Storage stability is essential for the commercial success of functional beverages (Kumar, Pipliya, Srivastav, & Srivastava, 2024). Factors such as pH, titratable acidity, syneresis, and microbial stability influence shelf life and consumer acceptance (Hussain et al., 2014). Retention of bioactive compounds, including phenolics, flavonoids, ascorbic acid, and anthocyanins, is critical for preserving health benefits over time (Fikry, Yusof, Al-Awaadh, Rahman, & Chin, 2020). Temperature significantly affects biochemical stability and microbial growth (Jadhav, Chavan, & Desale, 2014; Singh, Kaur, et al., 2024). Response surface methodology (RSM) is an effective tool for optimizing formulation and processing variables in lassi (Sharma et al., 2020; Sharma et al., 2021). By analyzing the interactions between microgreen concentration, milk content, and stabilizers, RSM helps identify optimal conditions for achieving desirable sensory, nutritional, and functional properties. Kinetic modeling further facilitates understanding of quality changes during storage and enables accurate shelf-life prediction at different temperatures (Kumar et al., 2024; Zhi et al., 2017).

This study aims to develop a radish sango microgreens-based lassi, optimizing its formulation using Response Surface Methodology (RSM) to enhance its nutritional and sensory attributes. The research evaluates the impact of microgreen concentration and fermentation variables on consumer acceptability, bioactive composition, and storage stability, providing novel insights into microgreen integration in dairy beverages. Kinetic modeling assesses the shelf life at 5 °C, 15 °C, and 25 °C, analyzing temperature-dependent degradation rates to predict stability using advanced reaction modeling techniques. By addressing the interplay between formulation, processing, and storage, this study establishes microgreens as functional ingredients in dairy applications, contributing to the advancement of health-conscious, commercially viable functional beverages.

## 2 Materials and methodology

### 2.1 Raw materials and chemical reagents

Toned milk (3.0 % fat/8.5 % SNF; Verka Taaza), powder sugar (Trust Classic, India), and Carboxymethyl Cellulose (CMC) (La Casa, India) were procured from the local market of Phagwara-144,411, India and Chr. Hansen Exact Dahi 2- Mat no.706272 (Freeze dried lactic culture for Direct Vat, Denmark) culture was used for preparation of Dahi. For the experimental study work, all required chemicals are procured from LobaChemie (Mumbai, India) and Sigma-Aldrich (Steinheim, Germany).

### 2.2 Microgreens cultivation and juice preparation

Radish sango seeds were cultivated in a cocopeat-based medium under controlled conditions. Seeds were pre-treated with 2.00 % (*w*/*v*) calcium hypochlorite solution, rinsed thrice with distilled water, soaked for 12 h at 25 ± 2 °C, drained, and sown at 0.29 g/m^2^. The growth environment condition was maintained at 20 ± 2 °C with 65 ± 2 % relative humidity. For the first 3-days, the seeds were kept in the dark, followed by exposure to LED light (450–455 nm, 8 W, PPFD 45 ± 2 μmol m^−2^ s^−1^, 12 h photoperiod) for 6-days. On the fourth day, a foliar spray containing 18 mM calcium chloride and 8 mM potassium nitrate was applied daily until 9-days.

Microgreens were harvested on the 9-days, washed with 0.50 % (*w*/*v*) citric acid, and air-dried. The cleaned microgreens were pressed using a masticating juicer (Refurbished- Philips Masticating Slow Juicer 150 W - HR1887/81, Jalandhar, Punjab), and the obtained juices from radish sango microgreens were collected in plastic flasks and stored in glass bottles for further use at 4 ± 1 °C.

### 2.3 Preparation of Dahi

Dahi was prepared following the method described by Rasane et al. (2017). Fresh standardized milk was heat treated to 90 °C for 15 min, rapidly cooled to room temperature with chilled water, and inoculated with 1.00 % (*v*/v) of a starter culture. The prepared mixtures were incubated at 37 °C for 4 to 5 h until complete coagulation (pH level 4.5), then cooled overnight at 4 ± 1 °C.

### 2.4 Experimental design

**Fig. 1:**
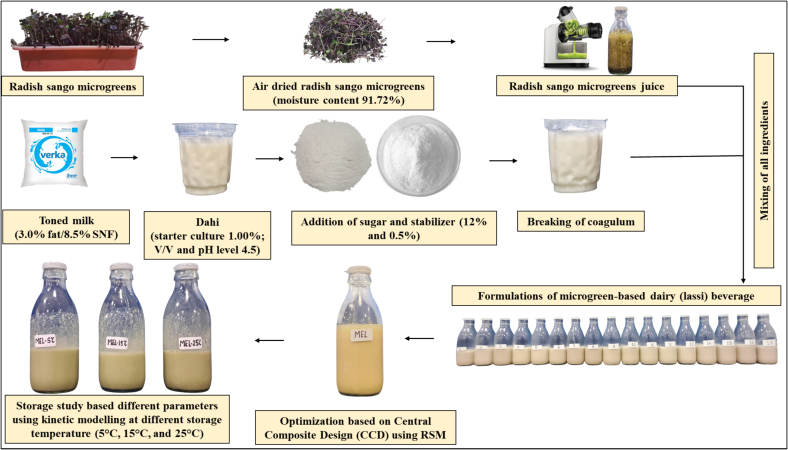
The different formulations of microgreens-based dairy (lassi) beverage using response surface methodology.

The preliminary study aimed to determine the optimal formulation of microgreens-based dairy (lassi) from a sensory perspective through trial-and-error testing. The development of lassi formulations was conducted using Design Expert software (version 13), a statistical analysis package for experimental modeling. Independent variables, including microgreen juice (A), dahi (B), and drinking water (C), were selected for formulation optimization. A total of 17 experimental runs were generated using the D-optimal model. The study explored the effects of these variables within specified percentage (%) ranges: microgreen juice (A) ranged from 5 % (−1) to 9.5 % (+1), dahi (B) from 60 % (−1) to 64.5 % (+1), and water (C) from 15 % (−1) to 19.5 % (+1) (supplementary table 1). Fixed concentrations of sugar (15 %) and CMC stabilizer (0.5 %) were maintained across all treatments. The experimental flow diagram illustrating the preparation process of lassi using response surface methodology (RSM) is shown in **(**Fig. 1**)**. The lassi was prepared following the method described by Kumar et al. (2023), with slight modifications **(SI** Fig. 1**)**. The prepared lassi samples were stored in 200 mL glass bottles at refrigeration conditions (4 ± 1 °C) for subsequent analyses. The data from dependent variables were analyzed using response surface regression analysis, applying the least squares methodology to fit the special cubic model. Model adequacy was assessed by evaluating lack of fit, coefficient of determination (R^2^), adequate precision, and F-values through analysis of variance (ANOVA).

### 2.5 Physicochemical properties

The pH of the beverage samples was measured at 20 °C using a digital pH meter (Model: BRPH-10, BR Biochem Life Sciences Pvt. Ltd., India) and calibration was performed using standard buffer solutions (pH 4.0 and pH 9.0). Titratable acidity (% lactic acid) was determined following Raje-Nimbalkar et al. (2023). Total soluble solids (TSS) were analyzed using a digital refractometer (Model: HI-96811, Hanna Instruments, India) at 20 °C, as described by Sharma et al. (2021).

Water holding capacity (WHC) was assessed using the method of Ahmed, Noor, Tariq, and Zaidi (2023) with modifications. A 5 *g* sample was centrifuged at 3282 ×*g* for 15 min at 20 °C, and the supernatant was discarded. WHC was calculated based on the residual precipitate weight using Eq. (01). Syneresis was evaluated by centrifuging samples at 3000 ×*g* for 15 min at 10 °C. The supernatant weight was recorded and expressed as a percentage of the initial sample weight, following Ghaderi-Ghahfarokhi, Shakarami, and Zarei (2024) using Eq. (02).

(01) $$\mathrm{WHC}\;\left(\%\right)=\frac{\text{Weight of sample}\;\left(g\right)-\text{Weight of sediments}\;\left(g\right)}{\text{Weight of sample}\;\left(g\right)}\times 100$$

(02) $$\mathrm{Syneresis}\;\left(\%\right)=\frac{\text{Weight of Supernatant}\;\left(g\right)}{\text{Weight of sample}\;\left(g\right)}\times 100$$

### 2.6 Bioactive compounds and antioxidant activities

Bioactive compounds and antioxidant activities, including total phenol content (TPC), and total flavonoid content (TFC) were analyzed using a modified method by Gunjal et al. (2024a). Extraction was performed by macerating 1 g of the sample in 10 mL of 80 % ethanol (80:20 *v*/v). The mixture was centrifuged at 6000 ×*g* for 15 min, and the supernatant was filtered, adjusted to 10 mL, and stored at 4 ± 1 °C for further analysis.

TPC was determined using the Folin-Ciocalteu method where the reaction absorbance was measured at 765 nm. Results were expressed as mg GAE/100 g fresh weight using a gallic acid calibration curve. The absorbance for reaction of TFC was recorded at 510 nm. TFC was expressed as mg QUE/100 g based on a quercetin standard curve.

DPPH radical scavenging activity was measured as per Xiao, Xu, Lu, and Liu (2020), absorbance was recorded at 517 nm to determine % DPPH inhibition was calculated using eq. 03. FRAP assay followed Xiao et al. (2020), absorbance was read at 593 nm. Antioxidant capacity was expressed as μmol TE/g using a Trolox calibration curve.

(03) $$\mathrm{DPPH Inhibition}\;\left(\%\right)=\left(\;\frac{\mathrm{Abs}.\text{control}\;\left(\mathrm{nm}\right)-\mathrm{Abs}.\text{sample}\;\left(\mathrm{nm}\right)}{\mathrm{Abs}.\text{control}\;\left(\mathrm{nm}\right)}\right)\times 100$$

### 2.7 Ascorbic acid and anthocyanin content

Ascorbic acid content was measured following Gunjal et al. (2024a). Well homogenized sample was mixed with 10 % trichloroacetic acid and Folin–Ciocalteu reagent. The resulted mixture was incubated for 20 min at room temperature, and absorbance was recorded at 760 nm. Ascorbic acid concentration was determined using an l-ascorbic acid calibration curve and expressed as mg/100 g.

Anthocyanin content was analyzed following Martínez-Ispizua et al. (2022). A 0.1 g sample was extracted in 10 mL of methanol-HCl-water solution (90:1:9), vortexed, incubated in the dark for 1 h, and centrifuged at 6000 ×*g* for 10 min. Absorbance was recorded at 534, 643, and 661 nm, and anthocyanin content was calculated as μmol/100 g.

(04) $$\mathrm{Anthocyanin content}\;\left(\mathrm{\mu mol}/100g\right)=(\left(0.0821\times \mathrm{Abs}.534\right)-\left(0.00687\times \mathrm{Abs}.643\right)-\left(0.002426\times \mathrm{Abs}.661\right)\times 5$$

### 2.8 Thiobarbituric acid and free fatty acid content

The thiobarbituric acid (TBA) and free fatty acid (FFA) content in samples were analyzed following Singh, Kaur, et al. (2024). For TBA content, 4 g of lassi was mixed with 100 mL of 20 % trichloroacetic acid and 100 mL of distilled water, left at room temperature for 10 min, and filtered through Whatman No. 1 paper. A 5 mL aliquot of filtrate was mixed with 5 mL of 10 mM thiobarbituric acid solution, incubated at 95 °C for 30 min, and absorbance was recorded at 532 nm.

For FFA content, 2 g of the sample was dissolved in 50 mL of ethanol in a 250 mL conical flask. A few drops of phenolphthalein indicator were added, and the solution was titrated with 0.10 N potassium hydroxide until a stable pink color persisted for 15 s. The FFA content was calculated using Eq. (06).

(05) TBAcontent=7.8×Abs.532nm

(06) $$\mathrm{FFA}\;\mathrm{value}\;\left(\mathrm{\mu g}/g\right)=\left(\frac{\text{Titrate value}\times \text{Normality of}\;\mathrm{KOH}\times 56.1}{\text{Weight of sample}\;\left(g\right)}\right)$$

where, 1 mL N/10 KOH = 0.028 g oleic acid.

### 2.9 Sensory properties

Sensory evaluation of the microgreen juice-based dairy (lassi) beverage samples was performed using a 9-point hedonic scale and descriptive analysis, assessing attributes such as color and appearance, flavor and sweetness, consistency, and overall acceptability. The sensory evaluation process for lassi beverage was approved by the Lovely Professional University Ethical Committee, reference number LPU/CA/024/13/05/14080. A panel of 30 semi-trained individuals (15 males and 15 females, aged 20 to 40 years) participated, with documented consent and understanding. The lassi samples were served in identical clear glass cups, labeled with random three-digit codes, and presented at a temperature of 7–10 °C to simulate typical consumption conditions (Singh, Singh, et al., 2024).

### 2.10 Microbial analysis

Microbial analysis, including total plate count and yeast and mold counts of the microgreen juice-based dairy (lassi) beverage sample, was conducted following the method by Kumar et al. (2023). A 1 g lassi sample was aseptically mixed with 9 mL of distilled water and thoroughly shaken to ensure homogenization. Serial dilutions were prepared, and samples were incubated at 37 °C for 24 to 48 h. The total plate count was determined on nutrient agar, while yeast and mold counts were assessed on potato dextrose agar. Results for total plate count, yeast and mold, and coliform counts were expressed as colony-forming units per milliliter (cfu/mL), based on colony counts from petri dishes.

### 2.11 Determination of shelf-life of optimized product

The shelf-life prediction of the product was carried using the three mathematical models i.e., zero, first and second order kinetics. The experimental data for all parameters was modeled and models fit was accessed using coefficient of determination (R^2^), chi-square (x^2^) and half-life (t_1/2_) of the compounds was estimated by using degradation rate constants.

#### 2.11.1 Establishment of the shelf-life model

The changes in overall acceptability, acidity (lactic acid %), FFA, TBA, and microbial parameters in optimized microgreen juice-based dairy beverage were studied under different storage temperature conditions at 5 °C, 15 °C, and 25 °C. Samples were analyzed at 3-days intervals to determine changes in selected parameters. The observed changes in the concentration of these compounds were modeled using zero-order, first-order, and second-order kinetics (Kumar et al., 2024), as shown in Eqs. (08, 09, and 10).

(08) $$C_{t}=C_{0}-k\times t$$

(09) $$C_{t}=C_{0}\times e^{-k\times t}$$

(10) $$\frac{1}{C_{t}}-\frac{1}{C_{0}}=k\times t$$

The temperature dependency of the k was modeled using the Arrhenius, Eyring, and Ball models, as shown in Eqs. (11, 13, and 14), respectively.

(11) $$\ln k=\ln k_{\mathrm{ref}}-{\frac{E_{a}}{R}}_{0}\left(\frac{1}{T}-\frac{1}{T_{\mathrm{ref}}}\right)$$

(12) $$T_{\mathrm{ref}}=\frac{1}{n}\sum_{i=0}^{n}T_{i}$$

(13) $$\ln\frac{k}{T}=-\frac{∆H}{R}\times \frac{1}{T}+\ln\frac{k_{B}}{h}+\frac{∆S}{R}$$

(14) $$\log_{10}\left(\frac{D}{D_{\mathrm{ref}}}\right)=-\left(\frac{T-T_{\mathrm{ref}}}{Z}\right)$$

(15) $$D=\frac{\ln10}{k}$$

where, △S represents the entropy of activation; △H represents the enthalpy of activation; h represents the Planck constant, 6.626 × 10^−34^ J s; kB represents the Boltzmann constant, 1.381 × 10^−23^ J/K; R represents the ideal gas constant, 8.314 J/(mol·K); T represents the absolute storage temperature (K), D represent the storage time taken by the quality index to reduce by 90 %, D_ref_ represents the D value at the reference temperature T_ref_, Z reparents the range of storage temperatures required the D value to reduce by 10 times (°C), T_ref_ represents the reference temperature (°C) (Eq. 12).

### 2.12 Cost estimation of beverage

The cost analysis of the optimized microgreen juice-based dairy (lassi) beverage was calculated by considering the expenses for raw materials, processing, and labor, including both fixed and variable costs, as outlined in the methodology described by Pandhi and Poonia (2021).

### 2.13 Statistical analysis

The mean values presented in this study were obtained from experiments conducted in triplicate. The optimal proportions of microgreen juice, dahi, and water for the development of microgreen juice-based dairy (lassi) beverage were determined using regression analysis and the Design Expert 13 software from Stat Ease Inc. in MN, USA. Multiple regression models were used for the analysis of the data, and ANOVA was utilized to determine the statistical significance of each outcome. Responses were collected based on the physicochemical, biochemical, and antioxidant parameters of the lassi beverage. We used SPSS Version 22.0 (IBM SPSS Inc., Armonk, NY, USA) to perform a two-factor analysis of variance on the data, after which we performed Tukey's pairwise comparison analysis. The kinetic analysis and data visualization were carried out using Microsoft Excel 2019 and OriginPro 9.0.

## 3 Results and discussion

### 3.1 Optimization of variables

To optimize the formulation of a functional fermented microgreens-based lassi beverage, response surface methodology was employed using a D-optimal mixture design. A total of 17 experimental runs (*n* = 3) were conducted to evaluate sensory, physicochemical, biochemical, and antioxidant parameters. The optimal composition was identified as 9.189 % microgreen juice, 60 % dahi, and 15.311 % water, combined with 15 % sugar and 0.50 % CMC stabilizer. The optimization criteria aimed to maximize microgreen juice (5–9.5 %) while minimizing dahi (60–64.5 %) and water (15–19.5 %) to achieve a balance of functional and sensory attributes. These criteria, detailed in **SI** Table 2, were designed to enhance product quality. The final formulation achieved a desirability score of 0.781, making it the best combination for this study, as shown in **SI** Fig. 2. Results, including observed and predicted responses, are presented in Table 2, validating the models reliability and the optimized formulation.

**Fig. 2:**
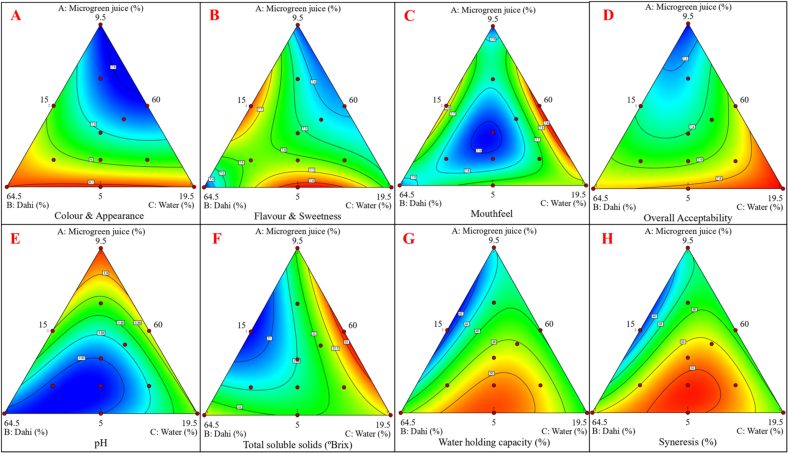
Response surface plots showing the effect of microgreen juice (A), dahi (B), and water (C) on microgreens-based dairy (lassi) beverage selected study parameters. Where, A: Color & Appearance; B: Flavor & Sweetness; C: Mouthfeel; D: Overall acceptability; E: pH; F: Total soluble solids; G: Water holding capacity; H: Syneresis.

### 3.2 Effect of processing variables on sensory parameters

The sensory parameters were significantly (P˂0.05) influenced by the proportions of key ingredients such as microgreen juice, dahi, and water which individually and in combination shaped consumer perceptions and preferences. Table 1a presents the sensory scores for various formulations, while Table 3 and **SI** Table 3 provide regression coefficients and F-values for polynomial models, confirming their adequacy with high R^2^ values (>90 %), reflecting strong predictive performance.

**Table 1a:** The effect of constituents on sensory parameters of microgreens-based dairy (lassi) beverage.

**Treatments**	**Dahi (%)**	**Microgreen extract (%)**	**Water (%)**	**Color & Appearance**	**Flavor & Sweetness**	**Consistency**	**Mouthfeel**	**Overall Acceptability**
1	60.00	9.50	15.00	7.81	7.35	7.87	7.52	7.05
2	60.75	5.75	18.00	7.97	7.56	7.88	7.68	7.73
3	60.75	8.00	15.75	7.83	7.48	7.82	7.61	7.24
4	60.00	5.00	19.50	8.09	7.52	7.94	7.75	7.96
5	62.25	5.00	17.25	8.11	7.84	7.97	7.76	7.85
6	62.25	7.25	15.00	7.98	7.79	7.75	7.88	7.33
7	60.00	9.50	15.00	7.81	7.29	7.89	7.48	7.05
8	60.00	7.25	17.25	7.82	7.39	7.85	7.94	7.68
9	63.00	5.75	15.75	8.02	7.66	7.91	7.56	7.54
10	60.00	5.00	19.50	8.09	7.56	7.94	7.79	7.96
11	64.50	5.00	15.00	8.12	7.29	8.07	7.49	7.77
12	61.88	5.75	16.88	7.99	7.62	7.89	7.53	7.57
13	64.50	5.00	15.00	8.08	7.29	8.11	7.60	7.77
14	62.25	5.00	17.25	8.12	7.84	7.97	7.76	7.85
15	60.75	6.88	16.88	7.85	7.43	7.83	7.63	7.47
16	62.25	7.25	15.00	7.98	7.79	7.79	7.83	7.39
17	61.50	6.50	16.50	7.91	7.51	7.84	7.48	7.42

Note: All the experimental results values are presented as mean value (*n* = 3) basis based on each sensory parameter.

The visual appeal, primarily dictated by microgreen juice concentration, resulted in color and appearance scores ranging from 7.81 to 8.12. Moderate juice concentrations yielded the highest scores (8.08–8.12), attributed to the subtle green tint blending harmoniously with the creamy white lassi base. Excess juice led to darker hues, less preferred by panelists, while low water content resulted in harsher tones. Regression analysis confirmed that linear and interaction effects of microgreen juice and water significantly (P˂0.05) impacted this parameter. Similar studies incorporating pudina extract (Dhumal, Padghan, Shinde, & Maske, 2018), ginger juice (Raje-Nimbalkar et al., 2023), and radish sango microgreens (Mlinarić et al., 2023) yielded comparable insights, highlighting the importance of color balance in dairy beverages.

Flavor and sweetness scores were strongly influenced by microgreen juice and water proportions. Radish sango microgreen juice imparted a subtle vegetal and mildly pungent flavor. However, at higher concentrations (9.5 %), the pungency overshadowed the inherent sweetness, lowering flavor scores to 7.29 due to phenolic-induced bitterness. Quadratic interactions between juice and water, shown in Table 3, revealed that moderate water dilution (18–19.5 %) reduced pungency and maintained sweetness. Similar trends were observed in mango and beetroot powder-enriched lassi (Chawla & Sivakumar, 2017), as well as other plant-based variants such as ginger, carrot, turmeric, and tomato pomace lassi (Kumar et al., 2023; Maji, Ray, & Ghatak, 2020). Optimization was necessary to balance functional benefits with consumer preferences.

The consistency and mouthfeel of the beverage were significantly affected by the dahi-to-water ratio. A smooth, creamy texture critical to dairy beverage was enhanced by higher dahi concentrations, resulting in improved consistency scores (8.07–8.11) (Gallardo-Escamilla, Kelly, & Delahunty, 2006). Excessive viscosity, however, led to a heavy mouthfeel, decreasing scores to 7.49–7.60. Regression analysis in Table 3 confirmed the significant (P˂0.05) linear effects of dahi and water proportions, while interactions shaped the final texture, as illustrated by the sensory response surface **(**Fig. 2C**)**. Similar findings were reported by Maji, Ray, Ghatak, and Chakraborty (2018) with turmeric extract in herbal lassi.

Overall acceptability, reflecting cumulative sensory attributes, ranged from 7.05 to 7.96 on a 9-point hedonic scale. As seen in Table 1a, moderate microgreen juice concentrations enhanced consumer preference, supported by response surface modeling **(**Fig. 2D**)**. The regression model (R^2^ = 99.82 %) accounted for total variations, emphasizing the role of balanced proportions. Studies on fenugreek and spinach microgreen juice-based beverages identified similar effects, where higher juice concentrations negatively impacted acceptability due to dark colors and vegetal flavors (Sharma et al., 2020; Sharma et al., 2021). For example, moringa-fortified lassi showed a reduction in overall acceptability scores from 8.50 to 6.25 with increased moringa powder (Mistry, Patel, Pinto, & Modha, 2018).

### 3.3 Effect of processing variables on physicochemical parameters

The physicochemical properties of the microgreen juice-based dairy (lassi) beverage, including pH, titratable acidity (lactic acid %), TSS, WHC, and syneresis, were significantly (*P* < 0.05) influenced by processing variables. These parameters are critical in dairy products, influencing microbial growth, flavor development, and milk protein coagulation, which are essential for fermented dairy products like cheese, dahi, and yogurt (Singh, Kaur, et al., 2024). Titratable acidity, expressed as lactic acid percentage, is vital for assessing fermentation quality and safety, while TSS contributes to texture and sweetness (Akhi, Ahmed, Ara, & Rana, 2023).

The pH of the microgreen juice-based dairy (lassi) beverage ranged from 3.81 to 3.92 across different formulations Table 1b. Both microgreen juice and dahi significantly (*P* < 0.05) influenced pH, while water had a diluting effect. Higher microgreen juice concentrations reduced pH due to the presence of organic acids, phenolics, and glucosinolates in radish sango microgreens (Kaur, Bhise, Kaur, & Minhas, 2018; Mlinarić et al., 2023). The formulation with 9.5 % microgreen juice had the lowest pH (3.81). Similarly, increasing dahi concentrations contributed to acidity due to lactic acid formation during fermentation (Akhi et al., 2023). Water addition mitigated acidification, leading to a relatively higher pH. A low pH enhances microbial stability but must be controlled to avoid excessive acidity, which can affect flavor. Response surface methodology (RSM) analysis **(**Fig. 2C**)** showed a significant (*P* < 0.05) impact of microgreen juice, dahi, and water on pH. The observed pH values align with previous studies on functional lassi beverages enriched with plant-based extracts (Kumar et al., 2023; Kumar, Hussain, Raju, Singh, & Singh, 2017).

**Table 1b:** The effect of constituents on physicochemical parameters of microgreens-based dairy (lassi) beverage.

**Treatments**	**Dahi (%)**	**Microgreen extract (%)**	**Water (%)**	**pH**	**Acidity (% lactic acid)**	**TSS (°Brix)**	**WHC (%)**	**Syneresis (%)**
1	60.00	9.50	15.00	3.91	0.58	22.42	43.69	45.66
2	60.75	5.75	18.00	3.84	0.55	22.29	49.66	51.78
3	60.75	8.00	15.75	3.87	0.57	21.51	44.91	46.95
4	60.00	5.00	19.50	3.88	0.55	22.52	45.93	47.95
5	62.25	5.00	17.25	3.82	0.55	21.96	51.86	52.88
6	62.25	7.25	15.00	3.87	0.57	20.74	41.05	42.05
7	60.00	9.50	15.00	3.92	0.58	21.88	44.05	46.05
8	60.00	7.25	17.25	3.89	0.56	23.17	45.82	47.22
9	63.00	5.75	15.75	3.81	0.56	21.48	48.12	50.18
10	60.00	5.00	19.50	3.88	0.55	22.58	45.93	47.95
11	64.50	5.00	15.00	3.81	0.55	22.53	47.22	49.22
12	61.88	5.75	16.88	3.81	0.56	21.63	50.66	52.66
13	64.50	5.00	15.00	3.82	0.54	22.51	47.22	50.22
14	62.25	5.00	17.25	3.82	0.55	21.96	50.22	51.36
15	60.75	6.88	16.88	3.85	0.57	22.19	47.99	49.99
16	62.25	7.25	15.00	3.89	0.58	20.74	41.25	43.25
17	61.50	6.50	16.50	3.82	0.56	21.52	48.82	50.88

Note: All the experimental results values are presented as mean value (n = 3) basis based on each physicochemical parameters. Where, TSS: Total soluble solid; WHC: Water holding capacity.

Titratable acidity ranged from 0.54 % to 0.58 % Table 1b. Higher microgreen juice levels increased acidity, consistent with the presence of organic acids (Kaur, Kumar, Goyal, Tanwar, & Kaur, 2018; Mlinarić et al., 2023; Sharma et al., 2020; Sharma et al., 2021). Similar findings were reported in mango soy yogurt (0.729 %) (Kumar & Mishra, 2004), tomato pomace-fortified lassi (0.65–0.78 %) (Kumar et al., 2023), and moringa pod powder-enriched lassi (0.24–0.65 %) (Mistry et al., 2018). Dahi significantly contributed to acidity due to its lactic acid content, with higher dahi concentrations leading to increased acidity. Water addition diluted acidity, reducing titratable acidity values. ANOVA analysis **SI** Table 3 indicated a special cubic model with an F-value of 20.74 and P < 0.05, demonstrating a significant influence of microgreen juice, dahi, and water on titratable acidity. The coefficient of determination (R^2^ = 0.9256) confirmed the model's reliability, and the final equation for titratable acidity is shown in Table 3.

TSS ranged from 20.74 to 23.17°Brix Table 1b, indicating the presence of sugars, minerals, and organic acids (Andrés, Tenorio, & Villanueva, 2014). Higher microgreen juice percentages increased TSS due to the presence of natural sugars and bioactive compounds **(**Fig. 2F**)**. The highest TSS (23.17°Brix) was recorded at 9.5 % microgreen juice. Dahi also significantly (*P* < 0.05) influenced TSS due to its lactose content, with formulations containing 64.5 % dahi showing slightly higher values (22.89°Brix). Water addition reduced TSS, with 19.5 % water yielding the lowest value (21.12°Brix). The final equation for TSS is presented in Table 3. ANOVA analysis **SI** Table 3 confirmed a significant special cubic model (F-value = 56.47, *P* < 0.05, R^2^ = 0.9713). Previous studies on plant-enriched lassi reported similar findings, such as pudina extract (11.16–11.55°Brix) (Dhumal et al., 2018), mango powder (9.80–17°Brix), and beetroot powder (9.80–11.30°Brix) (Chawla & Sivakumar, 2017).

WHC, which measures the beverage's ability to retain water and resist syneresis, ranged from 41.05 % to 51.86 %, while syneresis ranged from 42.05 % to 52.88 % Table 1b. Higher WHC ensures a creamy texture and prevents phase separation, while lower syneresis improves shelf life and consumer acceptance (Kumar et al., 2023; Kumar, Hussain, Prasad, Singh, & Singh, 2021). Increased microgreen juice percentages improved WHC and reduced syneresis due to bioactive compound binding (Sanyukta et al., 2023). Higher dahi levels significantly enhanced WHC and minimized syneresis by forming a cohesive protein network. These findings align with previous studies demonstrating the stabilizing effects of microgreens and dairy proteins in functional dairy beverages **(**Fig. 2G & H**)**. Overall, the physicochemical properties of the lassi beverage were significantly influenced by ingredient composition.

### 3.4 Effect of processing variables on biochemical and antioxidant parameters

#### 3.4.1 Ascorbic acid and anthocyanin content

Ascorbic acid, a key bioactive compound known for its antioxidant properties and immune-boosting benefits (Gunjal et al., 2024b; Singh, Kaur, et al., 2024), was significantly influenced by microgreen juice concentrations in the prepared lassi beverage. Its content ranged from 32.96 to 48.86 mg/100 g **(**Table 1c**)**, with higher microgreen juice percentages yielding increased levels of ascorbic acid. The regression model **(**Table 3**)** confirmed significant (*P* < 0.05) linear effects of microgreen juice, while dahi and water had minimal influence. Comparative studies have shown similar findings, such as lassi with 2.5 % orange peel powder achieving ascorbic acid levels of 136 mg/100 g (Ananthakumar & Narayanan, 2021) and yogurt fortified with *Mangifera indica* and *Myrciaria dubia* displaying ascorbic acid ranges of 57.3 to 115.1 mg/100 g (Renteria, Espinoza-Espinoza, Valdiviezo-Marcelo, & Moreno-Quispe, 2022).

**Table 1c:** The effect of constituents on biochemical and antioxidant parameters of microgreens-based dairy (lassi) beverage.

**Treatments**	**Dahi (%)**	**Microgreen extract (%)**	**Water (%)**	**Anthocyanin content (μmol/100** **g)**	**Total phenol content (mg GAE/100** **g)**	**Total flavonoid content (mg QUE/100** **g)**	**Antioxidant activity DPPH (%)**	**Antioxidant activity FRAP (μmol TE/g)**	**Ascorbic acid (mg/100** **g)**
1	60.00	9.50	15.00	36.55	42.23	23.43	59.48	8.84	48.86
2	60.75	5.75	18.00	25.88	33.97	13.61	54.11	5.94	34.98
3	60.75	8.00	15.75	32.62	41.76	19.10	58.29	7.38	42.65
4	60.00	5.00	19.50	23.79	29.83	11.98	51.91	5.41	32.96
5	62.25	5.00	17.25	24.91	28.88	12.03	51.82	5.78	34.08
6	62.25	7.25	15.00	33.55	39.85	17.04	56.78	6.67	41.18
7	60.00	9.50	15.00	36.68	42.23	23.43	59.48	8.84	48.86
8	60.00	7.25	17.25	38.68	45.87	17.11	56.62	6.51	38.89
9	63.00	5.75	15.75	24.89	32.28	13.41	54.04	6.06	36.20
10	60.00	5.00	19.50	23.73	29.83	11.98	51.91	5.41	32.96
11	64.50	5.00	15.00	24.42	30.78	11.44	51.66	5.61	34.32
12	61.88	5.75	16.88	22.55	31.53	13.58	54.55	6.15	35.76
13	64.50	5.00	15.00	24.44	30.79	12.54	51.33	5.60	34.35
14	62.25	5.00	17.25	24.25	29.35	11.95	51.88	5.78	34.02
15	60.75	6.88	16.88	28.69	39.23	16.53	55.77	6.59	38.36
16	62.25	7.25	15.00	33.18	37.59	16.57	56.66	6.22	42.28
17	61.50	6.50	16.50	24.18	35.13	15.97	55.55	6.51	37.68

Note: All the experimental results values are presented as mean value (n = 3) basis based on each biochemical and antioxidant parameter. Where, GAE: Gallic acid equivalent; QUE: Quercetin equivalent; DPPH: 2,2-Diphenyl-1-Picrylhydrazyl; FRAP: Ferric reducing antioxidant power.

**Fig. 3:**
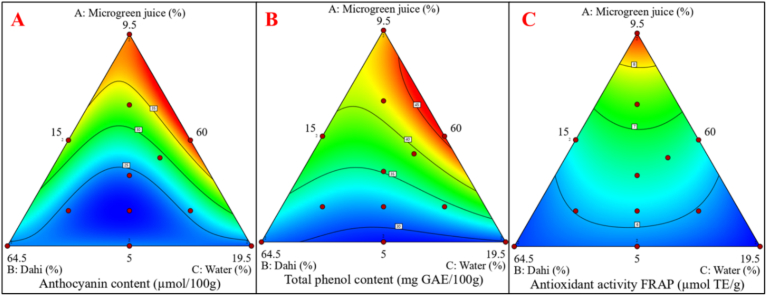
Response surface plots showing the effect of microgreen juice (A), dahi (B), and water (C) on microgreens-based dairy (lassi) beverage selected study parameters. Where, A: Anthocyanin content; B: Total phenol content; C: Antioxidant activity FRAP.

Anthocyanins, renowned for their antioxidant activity, are abundantly present in radish sango microgreens (Zhang et al., 2018) and significantly contribute to the beverage functional qualities. The anthocyanin content in lassi formulations ranged from 22.55 to 38.68 μmol/100 g **(**Table 1c**)**, with the highest levels observed in formulations containing 9.5 % microgreen juice. Regression analysis **(**Table 3**)** indicated significant (*P* < 0.05) linear and quadratic effects of microgreen juice, alongside interactions with dahi and water. Excessive water (19.5 %) diluted anthocyanin content, whereas lower water and higher juice concentrations enhanced retention. The response surface plot **(**Fig. 3A**)** further demonstrated a direct correlation between microgreen juice concentration and anthocyanin levels. These findings align with studies incorporating mulberry pomace powder (1–3 %), where anthocyanin content ranged from 23.29 to 79.09 g cyanidin-3-glucoside/g in fortified yogurt (Du et al., 2021).

#### 3.4.2 Total phenol and flavonoid content

Phenolic and flavonoid compounds are vital for antioxidant activity and contribute to the dairy (lassi) beverage functional properties. The TPC and TFC of the lassi beverage ranged from 28.88 to 45.87 mg GAE/100 g and 11.44 to 23.43 mg QUE/100 g were observed in developed lassi beverage samples, respectively Table 1c. Formulations with higher microgreen juice concentrations exhibited elevated TPC and TFC. This increase is attributed to the abundant phenolic compounds in radish sango microgreens, which are concentrated in the juice. The **(**Fig. 2B**)** depicts the relationship between TPC and the processing variables, showing that higher dahi and juice significantly (P < 0.05) increased TPC. The Table 3 shows positive significant (*P* < 0.05) linear and quadratic effects of microgreen juice and dahi on TPC and TFC. The regression model high R^2^ value (0.9943 and 0.9960) indicates strong predictive reliability. The statistical analysis using ANOVA **SI** Table 3 revealed that the special cubic model showed an F-value of 289.56 and 411.03, with a *P*-value of less than (P < 0.05), suggesting a significant model fit. Our results are consistent with findings from Alqahtani, Helal, Alnemr, and Marquez (2020), who observed a similar trend in TPC in yogurt with cranberry and tomato pomace. In another study, antioxidant-enriched herbal lassi incorporated with lemongrass, the TPC observed in formulated lassi is 50.28 mg GAE/100 g, respectively (Shendurse, Patel, Gopikrishna, Prajapati, & Gawai, 2024). Additionally, Maji et al. (2020) reported a TPC range of 0.124 to 0.226 mg GAE/g for various types of herbal lassi (ginger, carrot, turmeric), which was lower than our results. Numerous studies, including Gunjal et al. (2024b), have demonstrated that microgreens contain higher levels of bioactive compounds compared to their mature counterparts.

#### 3.4.3 Antioxidant activity

The antioxidant activities of the lassi were analyzed using the DPPH radical scavenging activity and FRAP. Both the antioxidant properties showed significant (*P* < 0.05) variation based on the processing variables of microgreen juice, dahi, and water. The DPPH and FRAP antioxidant activity ranged from 51.33 % to 59.48 % and 5.41 to 8.84 μmol TE/g, were observed respectively Table 1c. The lassi beverage formulations with higher microgreens juice (%) exhibited greater antioxidant properties. This trend aligns with the higher levels of anthocyanins, phenolics, and flavonoids in these formulations. Radish sango microgreens are rich in phenolics, flavonoids, anthocyanins, and ascorbic acid (Gunjal et al., 2024a; Singh, Singh, et al., 2024). Higher microgreen juice concentrations directly enhance the bioactive compound content and antioxidant activities (Sharma et al., 2020). However, excessive juice levels may lead to sensory challenges (Kaur, Kumar, et al., 2018). The regression model **SI** Table 2 confirmed significant linear and quadratic effects of juice concentration on the antioxidant activities of (DPPH and FRAP). The addition of water significantly reduced the activity by diluting the antioxidant compounds. The dahi provides a protective environment for bioactive compounds, reducing oxidative degradation of ascorbic acid and phenolic compounds. Moderate dahi enhances the solubility and stability of phenolics and flavonoids. The regression analysis Table 3 revealed significant interactions between microgreen juice and water, with optimal combinations enhancing the radical scavenging activity and ferric-reducing power of the lassi beverage. In **(**Fig. 3C**)** further illustrates the positive correlation between microgreen juice and the antioxidant activity of FRAP. The regression model for antioxidant activity of DPPH and FRAP high R^2^ value (0.9902 and 0.9902) indicates strong predictive reliability. The statistical analysis using ANOVA **SI** Table 3 revealed that the special cubic model showed an F-value of 269.78 and 270.73, with a P-value of less than (P < 0.05), suggesting both significant models fit. The DPPH scavenging activity of symbiotic lassi with honey was recorded at 28.43 % (Shah et al., 2016). Additionally, lassi enhanced with tomato pomace powder exhibited DPPH antioxidant activity ranging from 13.11 % to 28.12 % (Kumar et al., 2023). These values are somewhat lower compared to our findings. In another study, antioxidant-enriched herbal lassi incorporated with Lemongrass, antioxidant capacity such as DPPH and FRAP were observed in formulated lassi is 33.53 and 24.62 μM TE/100 g, respectively (Shendurse et al., 2024).

**Table 2:** Predicted and observed optimum responses for microgreens-based dairy (lassi) beverage.

**Responses**	**Predicted values**	**Observed values**
Microgreen juice (%)	9.189	
Dahi (%)	60	
Water (%)	15.311	
Color & Appearance	7.796	7.85 ± 0.45
Flavor & Sweetness	7.324	7.38 ± 0.26
Consistency	7.871	7.93 ± 0.35
Mouthfeel	7.598	7.78 ± 0.18
Overall Acceptability	7.156	8.29 ± 0.10
pH	3.911	4.35 ± 0.01
Acidity (% lactic acid)	0.58	0.62 ± 0.01
TSS (°Brix)	22.362	22.42 ± 0.08
WHC (%)	44.203	44.32 ± 0.17
Syneresis (%)	46.047	46.30 ± 0.36
Anthocyanin content (μmol/100 g)	37.828	37.91 ± 0.11
Total phenol content (mg GAE/100 g)	43.941	44.14 ± 0.28
Total flavonoid content (mg QUE/100 g)	22.489	22.62 ± 0.18
Antioxidant activity DPPH (%)	59.177	59.32 ± 0.20
Antioxidant activity FRAP (μmol TE/g)	8.447	8.30 ± 0.21
Ascorbic acid (mg/100 g)	47.228	59.32 ± 0.20

The obtained results from experiments are represented in mean ± standard deviation values with displayed significant differences *(P˂0.05)*. Where, TSS: Total soluble solid; WHC: Water holding capacity; GAE: Gallic acid equivalent; QUE: Quercetin equivalent; DPPH: 2,2-Diphenyl-1-Picrylhydrazyl; FRAP: Ferric reducing antioxidant power.

**Table 3:** The predicted models to experimental data in D-optimal design for the effect of microgreens-based dairy (lassi) beverage.

**Variables**	**Regression equation**	**Mean** **±** **SD**	**C·V. %**	**R**^**2**^	**Adjusted R**^**2**^	**Predicted R**^**2**^	**Adeq precision**
Color & Appearance	7.81 × A + 8.10 × B + 8.09 × C + 0.0982 × AB-0.5233 × AC + 0.0804 × BC-1.52 × ABC	7.98 ± 0.09	0.12	0.9957	0.9932	0.9848	49.12
Flavor & Sweetness	7.32 × A + 7.30 × B + 7.54 × C + 1.95 × AB-0.1775 × AC + 1.70 × BC-6.69 × ABC	7.54 ± 0.02	0.29	0.9917	0.9867	0.9749	38.56
Consistency	7.88 × A + 8.09 × B + 7.94 × C-0.8554 × AB-0.2395 × AC-0.1847 × BC + 0.2665 × ABC	7.90 ± 0.01	0.18	0.9844	0.9750	0.9480	33.50
Mouthfeel	7.50 × A + 7.54 × B + 7.77 × C + 1.32 × AB+1.25 × AC + 0.4053 × BC-12.13 × ABC	7.66 ± 0.03	0.41	0.9710	0.9537	0.8936	22.64
Overall Acceptability	7.05 × A + 7.77 × B + 7.96 × C-0.2073 × AB+0.6976 × AC-0.0605 × BC-6.29 × ABC	7.57 ± 0.11	0.20	0.9982	0.9971	0.9943	90.29
pH	3.91 × A + 3.81 × B + 3.88 × C + 0.0576 × AB-0.0229 × AC-0.1117 × BC-1.10 × ABC	3.85 ± 0.06	0.15	0.9833	0.9733	0.9399	26.70
Acidity	0.5835 × A + 0.5447 × B + 0.5476 × C + 0.0361 × AB-0.0165 × AC + 0.0155 × BC + 0.0307 × ABC	0.56 ± 0.04	0.82	0.9256	0.8810	0.7516	12.99
Total soluble solids	22.12 × A + 22.53 × B + 22.56 × C-6.43 × AB+3.26 × AC-2.28 × BC-7.75 × ABC	21.98 ± 0.14	0.63	0.9713	0.9541	0.9069	27.05
WHC	43.82 × A + 47.23 × B + 46.00 × C-17.65 × AB+3.56 × AC + 18.12 × BC + 76.62 × ABC	46.73 ± 0.42	0.91	0.9883	0.*812	0.9638	36.66
Syneresis	43.81 × A + 49.73 × B + 48.05 × C-20.61 × AB+1.27 × AC + 13.49 × BC + 107.83 × ABC	48.60 ± 0.57	1.49	0.9789	0.9662	0.9347	26.70
Anthocyanin content	36.55 × A + 24.52 × B + 23.77 × C + 11.45 × AB+33.51 × AC + 2.17 × BC-247.71 × ABC	28.41 ± 0.30	1.06	0.9981	0.9970	0.9940	82.86
Total phenol content	42.26 × A + 30.81 × B + 29.79 × C + 8.97 × AB+39.45 × AC-4.91 × BC-107.29 × ABC	35.36 ± 0.53	1.50	0.9943	0.9908	0.9800	49.33
Total flavonoid content	23.43 × A + 11.95 × B + 11.97 × C-3.74 × AB-2.32 × AC-0.2499 × BC + 13.41 × ABC	15.38 ± 0.30	1.99	0.9960	0.9935	0.9866	58.67
Antioxidant activity DPPH	59.49 × A + 51.51 × B + 51.92 × C + 5.01 × AB+3.08 × AC + 1.01 × BC + 12.02 × ABC	54.81 ± 0.27	0.50	0.9939	0.9902	0.9806	44.82
Antioxidant activity FRAP	8.85 × A + 5.61 × B + 5.40 × C-3.07 × AB-2.48 × AC + 1.09 × BC + 10.51 × ABC	6.43 ± 0.10	1.61	0.9939	0.9902	0.9785	51.95
Ascorbic acid	48.84 × A + 34.32 × B + 32.97 × C + 0.4387 × AB-8.04 × AC + 1.66 × BC-9.77 × ABC	38.14 ± 0.25	0.38	0.9984	0.9974	0.9944	95.35

Where, C.V: Coefficient of Variation; R^2^: Coefficient of Determination.

### 3.5 Effect of storage conditions on physicochemical properties

**Fig. 4a:**
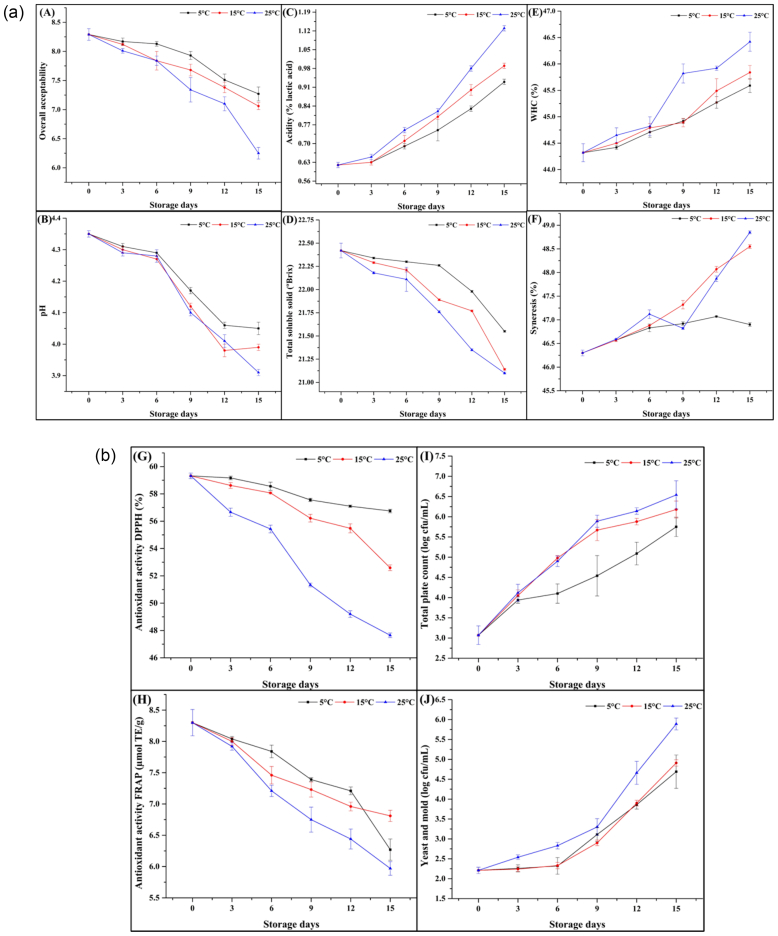
Effect of storage temperature conditions on physicochemical parameters of microgreens-based dairy (lassi) beverage. Where, A: Overall acceptability, B: pH, C: Acidity, D: Total soluble solids, E: Water holding capacity (WHC), F: Syneresis. 4b Effect of storage temperature conditions on antioxidant and microbial parameters of optimized microgreens-based dairy (lassi) beverage. Where, G: Antioxidant activity DPPH, H: Antioxidant activity FRAP, I: Total plate count, J: Yeast and mold count.

The pH of the dairy-based lassi consistently decreased during storage across all temperature conditions, indicating increased acidity due to microbial fermentation and chemical changes. On day 0, the pH value was observed to be 4.35 for all storage conditions. By the end of the 15-days storage period, the pH decreased to 4.05 at 5 °C, 3.99 at 15 °C, and 3.91 at 25 °C Fig. 4a **(A)**. Temperature significantly affected (P < 0.05) the pH value of the optimized lassi beverage, with higher storage temperatures accelerating the decline in pH (Ziarno, Zaręba, Ścibisz, & Kozłowska, 2023). At 25 °C, microbial activity was more pronounced, leading to the rapid production of lactic acid and a steeper drop in pH. In contrast, the lower temperature condition (5 °C) slowed microbial activity, maintaining a higher pH for a longer period (Łopusiewicz et al., 2019). This decreasing trend in pH value for the optimized lassi beverage aligns with increased microbial activity, as evidenced by total plate counts Fig. 4b **(I)**. Fermented dairy products like dahi contain naturally occurring lactic acid bacteria, which likely contributed to the acidification by fermenting residual sugars. The decrease in pH during storage is primarily attributed to the metabolic activity of lactic acid bacteria, which convert lactose into lactic acid (Pandhi & Poonia, 2021). The rate of acid production increases with temperature, consistent with the Arrhenius behavior of biochemical reactions. Additionally, interactions between microgreen juice and dairy proteins may contribute to the beverage buffering capacity, slowing the rate of pH decline at lower temperatures. Similarly, Krishna, Venkateshaiah, and Prabha (2019) observed a similar increase in the pH of probiotic lassi over a 12-days storage period. In another study, *Aloe vera*-supplemented synbiotic lassi exhibited a pH increase to 4.30–4.35 over a 16-days storage period (Kumar et al., 2021). Previous studies on fermented dairy products have also documented a reduction in pH with increasing storage temperatures (>10 °C), which aligns with our current observations (George, Arora, Sharma, Wadhwa, & Singh, 2010; Kumar et al., 2017).

The lactic acid content of the beverage increased over time, consistent with the observed pH trends. Initially, the optimized microgreen juice-based dairy (lassi) beverage contained 0.62 % lactic acid. By the end of the 15-days storage period, the lactic acid content increased to 0.93 % at 5 °C, 0.99 % at 15 °C, and 1.13 % at 25 °C Fig. 4a **(C)**. Similar to the pH trends, higher storage temperatures accelerated the increase in lactic acid formation. At 25 °C, the growth rate of lactic acid bacteria and enzymatic activity was higher, resulting in rapid acid production in the lassi beverage (Rawat, Kumari, Kumar, Ahlawat, & Sindhu, 2022). The increase in acidity negatively impacted sensory attributes, particularly flavor, and overall acceptability. Conversely, at 5 °C, the lower temperature effectively slowed the rate of acidification, preserving sensory quality for a longer period (Rani, Dabur, Bishnoi, & Jairath, 2023). The increase in acidity is primarily driven by the fermentation of lactose into lactic acid by lactic acid bacteria (Shah et al., 2016). A similar increasing trend was observed for titratable acidity in herbal honey lassi, which rose from 0.77 % to 1.38 % over a 28-days storage period (Kumar, Rai, & Kumari, 2020). The presence of bioactive compounds from radish sango microgreens, which may act as prebiotics, could also stimulate lactic acid bacteria growth, further influencing acidity (Lee, Lee, Kim, Shin, & Park, 2023).

TSS, which indicate the presence of dissolved solids such as sugars and minerals, remained relatively stable in the optimized lassi beverage during storage under different conditions Fig. 4a **(D)**. On the initial day, the TSS content was 22.42°Brix. By the 15-days of storage, the TSS content had decreased to a range of 21.10 to 21.55°Brix, with the decline being more pronounced at the higher temperature of 25 °C. This reduction could be attributed to the utilization of sugars by microbes during fermentation, which reduces the concentration of soluble solids in the lassi beverage (Silva, Silva, Costa-Lima, Carneiro, & Conte-Junior, 2017). The stability of TSS indicates that the beverage formulation maintained its structural integrity, with minimal breakdown of macronutrients such as proteins and polysaccharides during storage (Furlán, Padilla, & Campderros, 2011). Similar trends were observed for the whey-based papaya beverage (Saravanakumar & Manimegalai, 2001), whey-based fermented beverage prepared by blending using pineapple and strawberry juice (Kaur, Bhise, et al., 2018).

The effect of storage temperature on the WHC and syneresis of the optimized microgreen juice-based dairy (lassi) beverage is presented in Fig. 4a **(E and F)**. On the day 0, the WHC and syneresis were observed to be 44.32 % and 46.30 %, respectively, across all storage conditions. By the end of the 15-days storage period, these values increased to 45.92 % and 46.90 % at 5 °C, 45.84 % and 48.55 % at 15 °C, and 46.42 % and 48.85 % at 25 °C, respectively. Temperature had a significant (*P* < 0.05) effect on the WHC and syneresis of the optimized lassi beverage, with higher storage temperatures (25 °C) accelerating these trends. Higher temperature conditions led to a more rapid decline in WHC, indicating destabilization of the lassi beverage (Hussain et al., 2014). This destabilization is likely due to increased protein (casein) denaturation and whey separation at elevated temperatures. During this study, the optimized lassi beverage stored at 5 °C retained better WHC, maintaining the creamy texture of the beverage (Asaduzzaman, Mahomud, & Haque, 2021). The presence of stabilizers like CMC in the formulation likely mitigated these effects, particularly at lower temperatures (Kumar et al., 2021). Similarly, the increase in syneresis was more pronounced at higher temperatures, consistent with greater destabilization of the protein matrix and higher rates of microbial activity at elevated temperatures (Kumar et al., 2021). The increasing syneresis negatively impacted the texture and visual appeal of the beverage, making it less desirable to consumers.

The overall acceptability of the microgreen juice-based dairy (lassi) beverage, evaluated on a 9-point hedonic scale, varied significantly with storage temperature (5 °C, 15 °C, and 25 °C) over 15-days Fig. 4a **(A)**. On the initial day, the optimized formulation scored the highest acceptability (8.15), attributed to its balanced flavor, creamy texture, and fresh appearance. By the end of the 15-days storage period, these values decline to 7.27 at 5 °C, 7.06 at 15 °C, and 6.25 at 25 °C, respectively. Minimal changes in sensory attributes were noted, as refrigeration conditions (5 °C) effectively slowed biochemical and microbial activities. These results emphasize that refrigeration at 5 °C effectively preserves the lassi beverage sensory appeal for up to 15-days, while higher temperatures (15 °C and 25 °C) lead to accelerated degradation, reducing its consumer acceptability.

### 3.6 Effect of storage conditions on biochemical parameters

#### 3.6.1 Ascorbic acid and anthocyanin content

The ascorbic acid and anthocyanin content, key antioxidants in the microgreen juice-based dairy (lassi) beverage, declined over the storage period Table 4, with the rate of reduction significantly influenced by temperature (P < 0.05). The initial ascorbic acid content in the formulation was recorded as 62.85 mg/100 g, which declined progressively during storage at all temperatures. At 15-days, the retention was highest at 5 °C (56.75 mg/100 g), followed by 15 °C (51.65 mg/100 g) and 25 °C (47.65 mg/100 g). The loss of ascorbic acid was mainly attributed to oxidation reactions, which are enhanced under elevated temperature and oxygen exposure. Ascorbic acid is highly unstable and sensitive to both thermal and oxidative degradation during storage, especially in aqueous systems like beverages (Ahmed et al., 2023).

**Table 4:** Effect of storage temperature conditions on biochemical parameters of optimized microgreens-based dairy (lassi) beverage.

**Days**	**Temperature (°C)**	**Anthocyanin content (μmol/100** **g)**	**Total phenol content (mg GAE/100** **g)**	**Total flavonoid content (mg QUE/100** **g)**	**Ascorbic acid (mg/100** **g)**	**Free fatty acid content (μg/g)**	**Thiobarbituric acid content**
Day-0	5	37.91 ± 0.11^aA^	44.14 ± 0.28^aA^	22.62 ± 0.18^aA^	59.32 ± 0.20^aA^	0.42 ± 0.01^iC^	0.022 ± 0.00^jC^
	15	37.91 ± 0.11^aA^	44.14 ± 0.28^aB^	22.62 ± 0.18^aB^	59.32 ± 0.20^aB^	0.42 ± 0.01^iB^	0.022 ± 0.00^jB^
	25	37.91 ± 0.11^aB^	44.14 ± 0.28^aC^	22.62 ± 0.18^aB^	59.32 ± 0.20^aC^	0.46 ± 0.01^iA^	0.022 ± 0.00^jA^
Day-3	5	37.36 ± 0.09^abA^	44.27 ± 0.65^abA^	22.20 ± 0.08^abA^	59.17 ± 0.13^abA^	0.57 ± 0.01^hC^	0.029 ± 0.00^ijC^
	15	37.31 ± 0.16^abcA^	43.17 ± 0.08^bcdB^	22.10 ± 0.13^abB^	58.62 ± 0.21^abB^	0.59 ± 0.01^ghB^	0.034 ± 0.00^hiB^
	25	36.99 ± 0.18^bcB^	43.10 ± 0.13^cdeC^	22.25 ± 0.08^abB^	56.66 ± 0.30^efC^	0.63 ± 0.01^defghA^	0.036 ± 0.00^ghiA^
Day-6	5	37.07 ± 0.07^bcA^	43.76 ± 0.32^abcA^	21.93 ± 0.11^abcA^	58.56 ± 0.30^abcA^	0.60 ± 0.01^fghC^	0.037 ± 0.00^ghiC^
	15	37.09 ± 0.11^bcA^	42.43 ± 0.14^defB^	21.74 ± 0.22^bcdB^	58.07 ± 0.06^cdB^	0.67 ± 0.02^defB^	0.037 ± 0.00^ghiB^
	25	36.14 ± 0.17^defB^	42.42 ± 0.04^defC^	21.57 ± 0.23^bcdB^	55.43 ± 0.28^gC^	0.71 ± 0.02^cdA^	0.041 ± 0.00^efghA^
Day-9	5	36.67 ± 0.21^cdeA^	43.15 ± 0.02^cdA^	21.52 ± 0.37^bcdeA^	57.56 ± 0.12^cdA^	0.63 ± 0.01^efghC^	0.040 ± 0.00^fghC^
	15	36.79 ± 0.17^bcdA^	41.92 ± 0.13^fghB^	21.22 ± 0.05^cdeB^	56.22 ± 0.28^fgB^	0.70 ± 0.01^cdeB^	0.043 ± 0.00^defgB^
	25	35.96 ± 0.28^fgB^	42.09 ± 0.09^fgC^	21.02 ± 0.18^defgB^	51.33 ± 0.14^iC^	0.77 ± 0.03^bcA^	0.051 ± 0.00^bcdA^
Day-12	5	36.25 ± 0.18^defA^	42.55 ± 0.30^defA^	21.11 ± 0.05^defA^	57.10 ± 0.08^deA^	0.66 ± 0.01^defgC^	0.043 ± 0.00^defgC^
	15	36.09 ± 0.11^efA^	41.33 ± 0.27^ghB^	20.78 ± 0.16^efghB^	55.48 ± 0.33^gB^	0.78 ± 0.02^bcB^	0.048 ± 0.00^cdeB^
	25	35.31 ± 0.21^eB^	40.34 ± 0.15^iC^	20.32 ± 0.18^ghiB^	49.20 ± 0.24^jC^	0.84 ± 0.02^bA^	0.054 ± 0.00^bcA^
Day-15	5	35.39 ± 0.26^ghA^	42.15 ± 0.16^efgA^	20.42 ± 0.18^fghiA^	56.75 ± 0.13^defA^	0.77 ± 0.02^bcC^	0.047 ± 0.00^cdefC^
	15	35.19 ± 0.11^hA^	40.96 ± 0.14^hiB^	20.11 ± 0.06^hiB^	52.59 ± 0.21^hB^	0.84 ± 0.04^bB^	0.057 ± 0.00^abB^
	25	34.96 ± 0.14^hB^	40.19 ± 0.08^iC^	19.89 ± 0.36^iB^	47.65 ± 0.17^kC^	0.97 ± 0.02^aA^	0.065 ± 0.00^aA^

Note: The results were presented as mean ± standard deviations (n = 3). The different lowercase letters within the column indicate significant *(P* *<* *0.05)* differences among all treatments and different uppercase letters within the column indicate significant *(P* *<* *0.05)* differences in temperature conditions for the parameters. Where, GAE: Gallic acid equivalent; QUE: Quercetin equivalent.

Similarly, anthocyanins, which impart a distinct purple hue from Radish Sango microgreens and contribute to antioxidant potential, also showed a downward trend. The initial anthocyanin content of 36.49 μmol/100 g declined slightly to 35.39 μmol/100 g at 5 °C, 35.21 μmol/100 g at 15 °C, and 34.96 μmol/100 g at 25 °C by 15-days. Although the degradation was relatively low, it was still significant. The reduced degradation at lower temperatures suggests minimal structural changes to the anthocyanin molecules, which are known to be sensitive to pH, temperature, light, and oxygen (Mefleh et al., 2022). These pigments can undergo hydrolysis of glycosidic bonds and ring-opening reactions, reducing their stability and antioxidant efficacy. Thus, refrigerated storage conditions helped retain both the visual appeal and nutritional profile (Ścibisz et al., 2019).

#### 3.6.2 Total phenol and flavonoid content

The initial TPC was 44.14 mg GAE/100 g, and TFC was 22.62 mg QUE/100 g, observed on the initial day of storage. These bioactive components significantly (*P* < 0.05) decreased over the storage period, especially under elevated temperatures, Table 4. By 15-days, TPC decreased to 42.15 mg GAE/100 g at 5 °C, 41.26 mg at 15 °C, and 40.19 mg at 25 °C, while TFC dropped to 20.42, 20.01, and 19.89 mg QUE/100 g, respectively. The observed degradation in TFC and TFC can be attributed to both enzymatic oxidation (primarily due to polyphenol oxidase) and non-enzymatic oxidative reactions that are accelerated at higher storage temperatures. These reactions reduce antioxidant capacity by altering structural integrity and functional properties (Maji et al., 2018). Additionally, flavonoids are known to undergo hydroxylation, cleavage, and complex formation with proteins or other matrix components, contributing to their gradual decline during storage (Chung, Rojanasasithara, Mutilangi, & McClements, 2015).

The degradation rate is temperature-dependent, with faster losses at 25 °C, while cold storage at 5 °C effectively slows down oxidation and preserves flavonoid stability. These findings are in line with previous studies evaluating polyphenol retention in fortified dairy and fruit-based beverages during storage (Salazar-Orbea et al., 2023). Hence, refrigeration is recommended to maintain the nutritional integrity and functional quality of the lassi beverage during storage.

#### 3.6.3 Free fatty acid and thiobarbituric acid content

FFA content is an indicator of lipid hydrolysis, which occurs due to lipase or microbial activity and can affect taste, aroma, and safety. Initially, FFA levels were 0.42 μg/g, which increased significantly with storage. By 15-days, FFA values reached 0.77 μg/g (5 °C), 0.84 μg/g (15 °C), and 0.97 μg/g (25 °C) Table 4. Similarly, the TBA values, representing lipid peroxidation and secondary oxidation products such as malondialdehyde, rose from 0.022 mg MDA/kg (day 0) to 0.047, 0.057, and 0.065 mg MDA/kg at 5 °C, 15 °C, and 25 °C, respectively in Table 4.

The increase in FFA and TBA is associated with the oxidation of unsaturated lipids, especially in the presence of light, oxygen, and temperature fluctuations (Yadav et al., 2007). In microgreen powders and juice blends, bioactive compounds offer some oxidative resistance; however, lipid degradation can still occur. The results support earlier findings that suggested increased lipid oxidation in refrigerated dairy beverages beyond two weeks (Jain, Rasane, Jha, Sharma, & Gautam, 2014). Maintaining cold chain conditions is crucial for lipid stability and overall sensory acceptance.

#### 3.6.4 Antioxidant activity (DPPH and FRAP)

The DPPH free radical scavenging activity and ferric reducing antioxidant power (FRAP) are vital indicators of the beverage functional performance. At the start of storage, DPPH activity was 63.25 %, and FRAP value was 8.30 μmol TE/g. By 15-days, DPPH activity declined to 56.75 % at 5 °C, 50.25 % at 15 °C, and 47.65 % at 25 °C. Correspondingly, FRAP values reduced to 6.27, 6.81, and 5.97 μmol TE/g, respectively in Fig. 4b **(G and H)**.

The gradual decline in antioxidant activity reflects the loss of phenolics, flavonoids, anthocyanins, and ascorbic acid, which are primary contributors to redox potential (Firuzi, Lacanna, Petrucci, Marrosu, & Saso, 2004). The antioxidant capacity is tightly linked to the chemical structure and concentration of these compounds, and their breakdown over time limits the ability to neutralize reactive oxygen species. These observations are consistent with previous studies where temperature-sensitive degradation led to reduced functional attributes in herbal and fruit-enriched dairy beverages (Kumar & Kumar, 2016).

### 3.7 Effect of storage conditions on microbial parameters

Microbial quality is a critical aspect of the stability and safety of any food product, including dairy beverages like the microgreen juice-based dairy (lassi) beverage. The growth of microorganisms during storage can reduce the shelf life, sensory attributes, and safety of the beverage (Fusco et al., 2020). On the initial day, the total plate count was 3.07 log cfu/mL, which increased to 5.75 log cfu/mL at 5 °C, 6.18 log cfu/mL at 15 °C, and 6.54 log cfu/mL at 25 °C by 15-days, Fig. 4b **(I)**. The growth of yeasts and molds can lead to off-flavors, discoloration, and a decrease in product quality, making it essential to monitor their growth during storage (Sørhaug, 2011). The addition of different levels of orange juice significantly (P < 0.05) influenced the standard plate count (SPC) in the lassi samples during all the days of the storage period; the SPC count ranged from 5.99 to 7.34 × 10^7^ cfu/g over a 7-days storage period (Jadhav et al., 2014). In another study, the SPC count of turmeric lassi increased from 4.78 to 8.60 log cfu/mL over 9-days of storage (Maji et al., 2018). The observed differences in total plate count between storage conditions highlight the importance of temperature control in extending shelf life. At 5 °C, the microbial load remained within acceptable limits for dairy beverages, preserving both safety and sensory quality (Kumar et al., 2024). The total count values in the lassi beverage were lower than the 10^6^ cells/mL established by microbiological quality guidelines for ready-to-eat food (Fikry et al., 2020), suggesting that the optimized lassi is microbiologically stable during its shelf-life period. This stability might be due to the high content of antioxidants in the lassi, which can act as preservative agents.

Similarly, the yeast and mold count on the first day was 2.21 log cfu/mL, which increased to 4.69 log cfu/mL at 5 °C, 4.91 log cfu/mL at 15 °C, and 5.89 log cfu/mL at 25 °C by 15-days Fig. 4b **(J)**. The lower counts observed under refrigerated conditions indicate the efficacy of low-temperature storage in mitigating fungal contamination, thereby preserving the product's sensory and microbiological quality. Similar results were reported for different types of lassi beverages, including lassi prepared from orange juice (Jadhav et al., 2014), sweetened lassi (George et al., 2010), and symbiotic lassi containing honey (Sharma et al., 2015). The increase in total plate count and yeast and mold count with storage temperature can be attributed to the activation of microbial enzymes and metabolic processes that occur more rapidly at higher temperatures (Melaku, Satheesh, Awol, & Kassa, 2024). Refrigeration slows down microbial growth significantly by reducing the metabolic rates of bacteria.

### 3.8 Kinetic modeling of different parameters and shelf-life model

The reaction order for various quality indices, including overall acceptability, acidity, FFA, TBA, total plate count, and yeast and mold count, was determined by fitting experimental data to zero, first, and second-order reaction models. Among the models, the zero-order model exhibited the best fit (R^2^: 0.901–0.998), followed by the first-order model (R^2^: 0.862–0.993), and the second-order model (R^2^: 0.808–0.991) (Table 5). Consistent with previous research (Kumar et al., 2024; Singh, Kaur, et al., 2024), the zero-order model accurately described the changes in quality indices related to food deterioration kinetics.

**Table 5:** Reaction order estimation of quality indices based on the coefficient of determination (R^2^) from the zero-, first- and second-order reactions.

**Parameters**	**Temperature (°C)**	**Zero-order**			**First order**		**Second order**	
		**k**	**R**^**2**^	**t**_**1/2**_	**k**	**R**^**2**^	**k**	**R**^**2**^
Overall acceptability	5	0.0694	0.915	59.73	0.0089	0.909	0.0011	0.902
	10	0.0815	0.988	50.86	0.0106	0.983	0.0014	0.977
	25	0.1279	0.941	32.41	0.0175	0.920	0.0024	0.897
Acidity	5	0.0212	0.959	14.50	0.0283	0.973	0.0383	0.982
	10	0.0262	0.968	11.74	0.0339	0.976	0.0445	0.978
	25	0.0344	0.953	8.94	0.0413	0.979	0.0510	0.991
Free fatty acid	5	0.0191	0.898	10.99	0.0329	0.862	0.0583	0.808
	10	0.0255	0.945	8.24	0.0412	0.889	0.0690	0.819
	25	0.0305	0.970	6.89	0.0442	0.932	0.0664	0.868
Thiobarbituric acid	5	0.0016	0.960	6.72	0.0489	0.911	1.5344	0.845
	10	0.0021	0.970	5.12	0.0573	0.916	1.6530	0.822
	25	0.0027	0.972	3.98	0.0659	0.907	1.7848	0.798
Total plate count	5	0.1646	0.974	9.33	0.0382	0.961	0.0091	0.925
	10	0.2066	0.932	7.43	0.0451	0.888	0.0102	0.833
	25	0.2322	0.956	6.61	0.0491	0.915	0.0108	0.857
Yeast and mold	5	0.1715	0.888	6.43	0.0541	0.912	0.0178	0.922
	10	0.1816	0.854	6.07	0.0561	0.894	0.0181	0.920
	25	0.2403	0.901	4.59	0.6560	0.958	0.0191	0.989

Note: R^2^: coefficient of determination; k: rate of reaction; t_1/2_: half-life.

Reaction rate constants (k) demonstrated a significant impact of temperature on degradation kinetics, as absolute k values increased with rising temperatures, underscoring the importance of refrigeration. Storage temperature affected the degradation rates of key parameters like acidity, FFA, TBA, microbial growth, and sensory qualities, with rates being 3–6 times higher at 25 °C compared to 5 °C. These observations align with findings from previous studies on acidity, sensory evaluation (Zhi et al., 2017), and biochemical indices (Kumar & Mishra, 2004), emphasizing the necessity of low-temperature storage for maintaining product quality. To understand temperature sensitivity further, reaction rate constants were modeled using the Arrhenius, Eyring, and Ball eqs. **(SI** Table 4**)**. Activation energy (Ea) values obtained through the Arrhenius model ranged from 10.251 to 20.622 kJ/mol, with overall acceptability showing the highest Ea (20.622 kJ/mol), indicating its sensitivity to temperature fluctuations. Thermodynamic parameters derived from the Eyring model included negative entropy values (ΔS), suggesting a structured transition state during degradation, and activation enthalpy (ΔH), which aligned closely with Ea. These findings confirmed that the degradation process was non-spontaneous, and temperature driven.

The Ball model provided Dref and Z values for evaluating temperature sensitivity. Overall acceptability had the lowest *Z*-value (0.114 °C), indicating that minor temperature variations significantly impacted sensory degradation. Microbial parameters such as total plate count and yeast and mold count had higher Z-values (0.201 and 0.230), suggesting that microbial spoilage was less sensitive to temperature changes than sensory attributes. Nonetheless, significant Ea values for microbial indices underscored the need for low-temperature storage to restrict microbial growth. Using the Arrhenius equation **(**Table 5**)**, the shelf-life prediction for the optimized lassi beverage at 5 °C was 59.73, 14.50, 10.99, 6.72, 9.33, and 6.43 days (t_1/2_) for overall acceptability, acidity, FFA, TBA, total plate count, and yeast and mold count, respectively. Refrigeration effectively minimized degradation and microbial proliferation, extending the beverage's shelf life.

### 3.9 Optimized product cost

The raw material costs involved in preparing microgreens-based dairy (lassi) are detailed in **SI** Table 5. The production cost of the developed product aligns with current market trends, making it highly competitive with commercial lassi products. From an economic perspective, the product demonstrates high feasibility.

## 4 Conclusion

This study successfully optimized a radish sango microgreens-based lassi with enhanced bioactive compounds and antioxidant properties, using response surface methodology. The research provides novel insights into the development of a popular dairy beverage tailored for consumer health. Kinetic modeling revealed that storage at 5 °C preserved bioactive compounds and antioxidant activity and lowered microbial load after 15-days. Shelf-life analysis highlights the stability of bioactive compounds and antioxidant activity at low storage temperatures, demonstrating the beverage suitability for extended consumption. Furthermore, the product exhibits economic feasibility, aligning with market trends and showcasing its potential for commercialization. By combining nutritional richness, sensory appeal, and cost-effectiveness, this study bridges the gap between innovation and practicality, making significant contributions to the field of functional beverages.

## CRediT authorship contribution statement

**Mahendra Gunjal:** Writing – original draft. **Atul Khalangre:** Methodology, Investigation. **Jyoti Singh:** Visualization, Validation, Methodology. **Sezai Ercisli:** Writing – review & editing. **Prasad Rasane:** Conceptualization.

## Ethical Statement

This study involved sensory evaluation conducted with semi-trained panelists. The sensory evaluation process for the microgreens-based dairy (lassi) beverage was approved by the Lovely Professional University Ethical Committee, reference number LPU/CA/024/13/05/14080. Prior to participation, all participants were thoroughly informed about the study objectives, procedures, and potential risks. Written and/or verbal consent was obtained from each participant, who were also assured of their right to withdraw from the study at any time without any consequences. No personal data was collected or disclosed, ensuring confidentiality and privacy protection throughout the study. Additionally, no vulnerable populations were involved in this research.

## Funding

This research did not receive any specific grant from funding agencies in the public, commercial, or not-for-profit sectors.

## Declaration of competing interest

The authors declare that they have no known competing financial interests or personal relationships that could have appeared to influence the work reported in this paper.

## Data Availability

Data will be made available on request.

## Appendix A Supplementary data

Supplementary material Image 1
