# Supplementary material for: Novel approach for development and optimization of microgreens-based functional dairy beverage: Antioxidant enhancement, consumer acceptability, and kinetic shelf-life modeling
Source: Food Chem X. 2025 May 22;28:102559. doi: 10.1016/j.fochx.2025.102559 (PMC12159483; doi:10.1016/j.fochx.2025.102559)
Supplement: Supplementary file 1 — Supplementary material [file mmc1.docx]

**Supplementary information**

**Supplementary Figures**

**SI Fig. 1.** Flow chart for preparation of lassi using microgreen juice

| 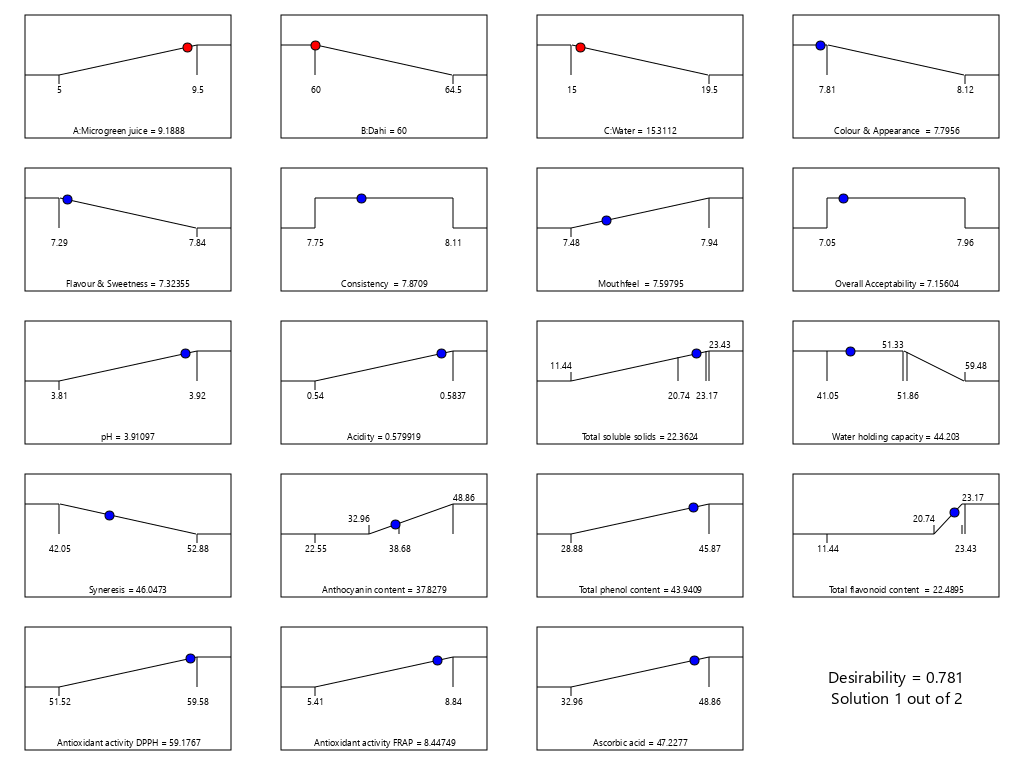 |
| --- |
| **SI Fig. 2.** Desirability ramp for optimization of microgreen juice-based dairy (lassi) beverage |

**Supplementary Tables**

**SI Table 1.** Experimental design optimization of levels of various constituents in microgreen juice-based dairy (lassi) using response surface methodology

| **Treatments** | **Dahi (%)** | **Microgreen extract (%)** | **Water (%)** | **Sugar (%)** | **CMC (%)** |
| --- | --- | --- | --- | --- | --- |
| 1 | 60.00 | 9.50 | 15.00 | 15.00 | 0.50 |
| 2 | 60.75 | 5.75 | 18.00 | 15.00 | 0.50 |
| 3 | 60.75 | 8.00 | 15.75 | 15.00 | 0.50 |
| 4 | 60.00 | 5.00 | 19.50 | 15.00 | 0.50 |
| 5 | 62.25 | 5.00 | 17.25 | 15.00 | 0.50 |
| 6 | 62.25 | 7.25 | 15.00 | 15.00 | 0.50 |
| 7 | 60.00 | 9.50 | 15.00 | 15.00 | 0.50 |
| 8 | 60.00 | 7.25 | 17.25 | 15.00 | 0.50 |
| 9 | 63.00 | 5.75 | 15.75 | 15.00 | 0.50 |
| 10 | 60.00 | 5.00 | 19.50 | 15.00 | 0.50 |
| 11 | 64.50 | 5.00 | 15.00 | 15.00 | 0.50 |
| 12 | 61.88 | 5.75 | 16.88 | 15.00 | 0.50 |
| 13 | 64.50 | 5.00 | 15.00 | 15.00 | 0.50 |
| 14 | 62.25 | 5.00 | 17.25 | 15.00 | 0.50 |
| 15 | 60.75 | 6.88 | 16.88 | 15.00 | 0.50 |
| 16 | 62.25 | 7.25 | 15.00 | 15.00 | 0.50 |
| 17 | 61.50 | 6.50 | 16.50 | 15.00 | 0.50 |

**SI Table 2.** Optimization criteria for different process and response variables for microgreen-based microgreen juice-based diary (lassi) beverage

| **Name** | **Goal** | **Lower Limit** | **Upper Limit** | **Lower Weight** | **Upper Weight** | **Importance** |
| --- | --- | --- | --- | --- | --- | --- |
| A: Microgreen juice | Maximize | 5 | 9.5 | 1 | 1 | 5 |
| B: Dahi | Minimize | 60 | 64.5 | 1 | 1 | 5 |
| C: Water | Minimize | 15 | 19.5 | 1 | 1 | 5 |
| Colour & Appearance | Minimize | 7.81 | 8.12 | 1 | 1 | 5 |
| Flavour & Sweetness | Minimize | 7.29 | 7.84 | 1 | 1 | 5 |
| Consistency | In range | 7.75 | 8.11 | 1 | 1 | 3 |
| Mouthfeel | Maximize | 7.48 | 7.94 | 1 | 1 | 5 |
| Overall Acceptability | In range | 7.05 | 7.96 | 1 | 1 | 5 |
| pH | Maximize | 3.81 | 3.92 | 1 | 1 | 3 |
| Acidity | Maximize | 0.54 | 0.5837 | 1 | 1 | 5 |
| Total soluble solids | Maximize | 20.74 | 23.17 | 1 | 1 | 5 |
| WHC | Minimize | 41.05 | 51.86 | 1 | 1 | 5 |
| Syneresis | Minimize | 42.05 | 52.88 | 1 | 1 | 5 |
| Anthocyanin content | Maximize | 22.55 | 38.68 | 1 | 1 | 5 |
| Total phenol content | Maximize | 28.88 | 45.87 | 1 | 1 | 5 |
| Total flavonoid content | Maximize | 11.44 | 23.43 | 1 | 1 | 5 |
| Antioxidant activity DPPH | Maximize | 51.33 | 59.48 | 1 | 1 | 5 |
| Antioxidant activity FRAP | Maximize | 5.41 | 8.84 | 1 | 1 | 5 |
| Ascorbic acid | Maximize | 32.96 | 48.86 | 1 | 1 | 5 |

**SI Table 3.** ANOVA special cubic model for the different responses

| **Responses** | **Source** | **Sum of Squares** | **df** | **Mean Square** | **F-value** | **P-value** | |
| --- | --- | --- | --- | --- | --- | --- | --- |
| Colour & Appearance | Model | 0.2185 | 6 | 0.0364 | 389.24 | < 0.0001 | significant |
|  | ⁽¹⁾Linear Mixture | 0.1801 | 2 | 0.09 | 962.36 | < 0.0001 |  |
|  | AB | 0.0008 | 1 | 0.0008 | 8.69 | 0.0146 |  |
|  | AC | 0.0151 | 1 | 0.0151 | 161.39 | < 0.0001 |  |
|  | BC | 0.0006 | 1 | 0.0006 | 5.95 | 0.0348 |  |
|  | ABC | 0.0034 | 1 | 0.0034 | 36.87 | 0.0001 |  |
|  | Residual | 0.0009 | 10 | 0.0001 |  |  |  |
|  | Lack of Fit | 0.0001 | 5 | 0 | 0.1006 | 0.9876 | not significant |
|  | Pure Error | 0.0008 | 5 | 0.0002 |  |  |  |
|  | Cor Total | 0.2194 | 16 |  |  |  |  |
| Flavour & Sweetness | Model | 0.5848 | 6 | 0.0975 | 199.09 | < 0.0001 | significant |
|  | ⁽¹⁾Linear Mixture | 0.062 | 2 | 0.031 | 63.36 | < 0.0001 |  |
|  | AB | 0.3213 | 1 | 0.3213 | 656.43 | < 0.0001 |  |
|  | AC | 0.0017 | 1 | 0.0017 | 3.55 | 0.089 |  |
|  | BC | 0.2504 | 1 | 0.2504 | 511.43 | < 0.0001 |  |
|  | ABC | 0.0671 | 1 | 0.0671 | 137.07 | < 0.0001 |  |
|  | Residual | 0.0049 | 10 | 0.0005 |  |  |  |
|  | Lack of Fit | 0.0023 | 5 | 0.0005 | 0.8827 | 0.5528 | not significant |
|  | Pure Error | 0.0026 | 5 | 0.0005 |  |  |  |
|  | Cor Total | 0.5896 | 16 |  |  |  |  |
| Consistency | Model | 0.1374 | 6 | 0.0229 | 105.07 | < 0.0001 | significant |
|  | ⁽¹⁾Linear Mixture | 0.0621 | 2 | 0.031 | 142.44 | < 0.0001 |  |
|  | AB | 0.0617 | 1 | 0.0617 | 282.9 | < 0.0001 |  |
|  | AC | 0.0032 | 1 | 0.0032 | 14.52 | 0.0034 |  |
|  | BC | 0.0029 | 1 | 0.0029 | 13.48 | 0.0043 |  |
|  | ABC | 0.0001 | 1 | 0.0001 | 0.4889 | 0.5004 |  |
|  | Residual | 0.0022 | 10 | 0.0002 |  |  |  |
|  | Lack of Fit | 0.0004 | 5 | 0.0001 | 0.2108 | 0.9437 | not significant |
|  | Pure Error | 0.0018 | 5 | 0.0004 |  |  |  |
|  | Cor Total | 0.1396 | 16 |  |  |  |  |
| Mouthfeel | Model | 0.3379 | 6 | 0.0563 | 55.87 | < 0.0001 | significant |
|  | ⁽¹⁾Linear Mixture | 0.049 | 2 | 0.0245 | 24.28 | 0.0001 |  |
|  | AB | 0.1469 | 1 | 0.1469 | 145.71 | < 0.0001 |  |
|  | AC | 0.0858 | 1 | 0.0858 | 85.14 | < 0.0001 |  |
|  | BC | 0.0142 | 1 | 0.0142 | 14.04 | 0.0038 |  |
|  | ABC | 0.2208 | 1 | 0.2208 | 219.01 | < 0.0001 |  |
|  | Residual | 0.0101 | 10 | 0.001 |  |  |  |
|  | Lack of Fit | 0.0012 | 5 | 0.0002 | 0.1327 | 0.9776 | not significant |
|  | Pure Error | 0.0089 | 5 | 0.0018 |  |  |  |
|  | Cor Total | 0.348 | 16 |  |  |  |  |
| Overall Acceptability | Model | 1.35 | 6 | 0.2249 | 904.5 | < 0.0001 | significant |
|  | ⁽¹⁾Linear Mixture | 1.25 | 2 | 0.6262 | 2519 | < 0.0001 |  |
|  | AB | 0.0036 | 1 | 0.0036 | 14.56 | 0.0034 |  |
|  | AC | 0.0268 | 1 | 0.0268 | 107.94 | < 0.0001 |  |
|  | BC | 0.0003 | 1 | 0.0003 | 1.27 | 0.2861 |  |
|  | ABC | 0.0593 | 1 | 0.0593 | 238.68 | < 0.0001 |  |
|  | Residual | 0.0025 | 10 | 0.0002 |  |  |  |
|  | Lack of Fit | 0.0007 | 5 | 0.0001 | 0.3811 | 0.8434 | not significant |
|  | Pure Error | 0.0018 | 5 | 0.0004 |  |  |  |
|  | Cor Total | 1.35 | 16 |  |  |  |  |
| pH | Model | 0.0222 | 6 | 0.0037 | 98.28 | < 0.0001 | significant |
|  | ⁽¹⁾Linear Mixture | 0.0159 | 2 | 0.0079 | 210.84 | < 0.0001 |  |
|  | AB | 0.0003 | 1 | 0.0003 | 7.42 | 0.0214 |  |
|  | AC | 0 | 1 | 0 | 0.7698 | 0.4009 |  |
|  | BC | 0.0011 | 1 | 0.0011 | 28.51 | 0.0003 |  |
|  | ABC | 0.0018 | 1 | 0.0018 | 48.22 | < 0.0001 |  |
|  | Residual | 0.0004 | 10 | 0 |  |  |  |
|  | Lack of Fit | 0.0001 | 5 | 0 | 0.2556 | 0.9197 | not significant |
|  | Pure Error | 0.0003 | 5 | 0.0001 |  |  |  |
|  | Cor Total | 0.0226 | 16 |  |  |  |  |
| Acidity | Model | 0.0027 | 6 | 0.0004 | 20.74 | < 0.0001 | significant |
|  | ⁽¹⁾Linear Mixture | 0.0025 | 2 | 0.0013 | 58.34 | < 0.0001 |  |
|  | AB | 0.0001 | 1 | 0.0001 | 5.08 | 0.0479 |  |
|  | AC | 0 | 1 | 0 | 0.696 | 0.4236 |  |
|  | BC | 0 | 1 | 0 | 0.9607 | 0.3501 |  |
|  | ABC | 0.000001412 | 1 | 1.412E-06 | 0.0654 | 0.8033 |  |
|  | Residual | 0.0002 | 10 | 0 |  |  |  |
|  | Lack of Fit | 0.0001 | 5 | 0 | 0.5872 | 0.7134 | not significant |
|  | Pure Error | 0.0001 | 5 | 0 |  |  |  |
|  | Cor Total | 0.0029 | 16 |  |  |  |  |
| Total soluble solids | Model | 6.68 | 6 | 1.11 | 56.47 | < 0.0001 | significant |
|  | ⁽¹⁾Linear Mixture | 1.34 | 2 | 0.6683 | 33.9 | < 0.0001 |  |
|  | AB | 3.48 | 1 | 3.48 | 176.63 | < 0.0001 |  |
|  | AC | 0.5874 | 1 | 0.5874 | 29.8 | 0.0003 |  |
|  | BC | 0.4474 | 1 | 0.4474 | 22.7 | 0.0008 |  |
|  | ABC | 0.09 | 1 | 0.09 | 4.57 | 0.0583 |  |
|  | Residual | 0.1971 | 10 | 0.0197 |  |  |  |
|  | Lack of Fit | 0.0493 | 5 | 0.0099 | 0.3336 | 0.8732 | not significant |
|  | Pure Error | 0.1478 | 5 | 0.0296 |  |  |  |
|  | Cor Total | 6.88 | 16 |  |  |  |  |
| WHC | Model | 153.21 | 6 | 25.54 | 140.49 | < 0.0001 | significant |
|  | ⁽¹⁾Linear Mixture | 60.53 | 2 | 30.27 | 166.52 | < 0.0001 |  |
|  | AB | 26.25 | 1 | 26.25 | 144.42 | < 0.0001 |  |
|  | AC | 0.7002 | 1 | 0.7002 | 3.85 | 0.0781 |  |
|  | BC | 28.27 | 1 | 28.27 | 155.55 | < 0.0001 |  |
|  | ABC | 8.81 | 1 | 8.81 | 48.45 | < 0.0001 |  |
|  | Residual | 1.82 | 10 | 0.1818 |  |  |  |
|  | Lack of Fit | 0.388 | 5 | 0.0776 | 0.2714 | 0.9107 | not significant |
|  | Pure Error | 1.43 | 5 | 0.2859 |  |  |  |
|  | Cor Total | 155.03 | 16 |  |  |  |  |
| Syneresis | Model | 153.99 | 6 | 25.66 | 77.19 | < 0.0001 | significant |
|  | ⁽¹⁾Linear Mixture | 60.48 | 2 | 30.24 | 90.95 | < 0.0001 |  |
|  | AB | 35.81 | 1 | 35.81 | 107.69 | < 0.0001 |  |
|  | AC | 0.0885 | 1 | 0.0885 | 0.2661 | 0.6171 |  |
|  | BC | 15.69 | 1 | 15.69 | 47.17 | < 0.0001 |  |
|  | ABC | 17.44 | 1 | 17.44 | 52.45 | < 0.0001 |  |
|  | Residual | 3.33 | 10 | 0.3325 |  |  |  |
|  | Lack of Fit | 0.8738 | 5 | 0.1748 | 0.3565 | 0.859 | not significant |
|  | Pure Error | 2.45 | 5 | 0.4903 |  |  |  |
|  | Cor Total | 157.31 | 16 |  |  |  |  |
| Anthocyanin content | Model | 481.7 | 6 | 80.28 | 887.16 | < 0.0001 | significant |
|  | ⁽¹⁾Linear Mixture | 368.72 | 2 | 184.36 | 2037.2 | < 0.0001 |  |
|  | AB | 11.05 | 1 | 11.05 | 122.06 | < 0.0001 |  |
|  | AC | 61.92 | 1 | 61.92 | 684.28 | < 0.0001 |  |
|  | BC | 0.4039 | 1 | 0.4039 | 4.46 | 0.0608 |  |
|  | ABC | 92.04 | 1 | 92.04 | 1017.1 | < 0.0001 |  |
|  | Residual | 0.9049 | 10 | 0.0905 |  |  |  |
|  | Lack of Fit | 0.6082 | 5 | 0.1216 | 2.05 | 0.2248 | not significant |
|  | Pure Error | 0.2967 | 5 | 0.0593 |  |  |  |
|  | Cor Total | 482.61 | 16 |  |  |  |  |
| Total phenol content | Model | 490.37 | 6 | 81.73 | 289.54 | < 0.0001 | significant |
|  | ⁽¹⁾Linear Mixture | 391.66 | 2 | 195.83 | 693.76 | < 0.0001 |  |
|  | AB | 6.79 | 1 | 6.79 | 24.04 | 0.0006 |  |
|  | AC | 85.8 | 1 | 85.8 | 303.97 | < 0.0001 |  |
|  | BC | 2.08 | 1 | 2.08 | 7.36 | 0.0218 |  |
|  | ABC | 17.27 | 1 | 17.27 | 61.17 | < 0.0001 |  |
|  | Residual | 2.82 | 10 | 0.2823 |  |  |  |
|  | Lack of Fit | 0.1584 | 5 | 0.0317 | 0.0595 | 0.9962 | not significant |
|  | Pure Error | 2.66 | 5 | 0.5329 |  |  |  |
|  | Cor Total | 493.2 | 16 |  |  |  |  |
| Total flavonoid content | Model | 231.56 | 6 | 38.59 | 411.03 | < 0.0001 | significant |
|  | ⁽¹⁾Linear Mixture | 230.19 | 2 | 115.09 | 1225.8 | < 0.0001 |  |
|  | AB | 1.18 | 1 | 1.18 | 12.58 | 0.0053 |  |
|  | AC | 0.2969 | 1 | 0.2969 | 3.16 | 0.1057 |  |
|  | BC | 0.0054 | 1 | 0.0054 | 0.0573 | 0.8157 |  |
|  | ABC | 0.2697 | 1 | 0.2697 | 2.87 | 0.121 |  |
|  | Residual | 0.9389 | 10 | 0.0939 |  |  |  |
|  | Lack of Fit | 0.2229 | 5 | 0.0446 | 0.3113 | 0.887 | not significant |
|  | Pure Error | 0.716 | 5 | 0.1432 |  |  |  |
|  | Cor Total | 232.5 | 16 |  |  |  |  |
| Antioxidant activity DPPH | Model | 124.54 | 6 | 20.76 | 269.78 | < 0.0001 | significant |
|  | ⁽¹⁾Linear Mixture | 119.52 | 2 | 59.76 | 776.74 | < 0.0001 |  |
|  | AB | 2.11 | 1 | 2.11 | 27.47 | 0.0004 |  |
|  | AC | 0.5216 | 1 | 0.5216 | 6.78 | 0.0263 |  |
|  | BC | 0.0871 | 1 | 0.0871 | 1.13 | 0.3124 |  |
|  | ABC | 0.2167 | 1 | 0.2167 | 2.82 | 0.1242 |  |
|  | Residual | 0.7694 | 10 | 0.0769 |  |  |  |
|  | Lack of Fit | 0.7059 | 5 | 0.1412 | 11.13 | 0.0097 | significant |
|  | Pure Error | 0.0635 | 5 | 0.0127 |  |  |  |
|  | Cor Total | 125.31 | 16 |  |  |  |  |
| Antioxidant activity FRAP | Model | 17.31 | 6 | 2.89 | 270.73 | < 0.0001 | significant |
|  | ⁽¹⁾Linear Mixture | 15.99 | 2 | 7.99 | 750.18 | < 0.0001 |  |
|  | AB | 0.7962 | 1 | 0.7962 | 74.71 | < 0.0001 |  |
|  | AC | 0.3396 | 1 | 0.3396 | 31.87 | 0.0002 |  |
|  | BC | 0.1022 | 1 | 0.1022 | 9.59 | 0.0113 |  |
|  | ABC | 0.1657 | 1 | 0.1657 | 15.55 | 0.0028 |  |
|  | Residual | 0.1066 | 10 | 0.0107 |  |  |  |
|  | Lack of Fit | 0.0053 | 5 | 0.0011 | 0.0525 | 0.9971 | not significant |
|  | Pure Error | 0.1013 | 5 | 0.0203 |  |  |  |
|  | Cor Total | 17.42 | 16 |  |  |  |  |
| Ascorbic acid | Model | 412.2 | 6 | 68.7 | 1021.3 | < 0.0001 | significant |
|  | ⁽¹⁾Linear Mixture | 405.67 | 2 | 202.83 | 3015.3 | < 0.0001 |  |
|  | AB | 0.0162 | 1 | 0.0162 | 0.2411 | 0.634 |  |
|  | AC | 3.56 | 1 | 3.56 | 52.93 | < 0.0001 |  |
|  | BC | 0.2363 | 1 | 0.2363 | 3.51 | 0.0904 |  |
|  | ABC | 0.1432 | 1 | 0.1432 | 2.13 | 0.1753 |  |
|  | Residual | 0.6727 | 10 | 0.0673 |  |  |  |
|  | Lack of Fit | 0.0654 | 5 | 0.0131 | 0.1078 | 0.9856 | not significant |
|  | Pure Error | 0.6073 | 5 | 0.1215 |  |  |  |
|  | Cor Total | 412.87 | 16 |  |  |  |  |

**SI Table 4.** Kinetic parameters of quality indices during storage obtained by the Arrhenius, Eyring, and Ball models.

| **Parameters** | **Arrhenius model** | | **Eyring models** | | | **Ball model** | | |
| --- | --- | --- | --- | --- | --- | --- | --- | --- |
|  | **Ea (kJ/mol)** | **R^2^** | **△H (kJ/mol)** | **△S (J/(mol.k)** | **R^2^** | **Z (°C)** | **D_ref_ (day)** | **R^2^** |
| Overall acceptability | 20.622 | 1.000 | 18.275 | -197.759 | 1.000 | 0.114 | 12.270 | 1.000 |
| Acidity | 15.490 | 0.963 | 13.145 | -201.174 | 0.948 | 0.152 | 38.168 | 0.960 |
| Free fatty acid | 14.143 | 0.847 | 11.797 | -201.837 | 0.793 | 0.166 | 39.216 | 0.842 |
| Thiobarbituric acid | 16.318 | 0.918 | 13.972 | -203.369 | 0.891 | 0.144 | 476.190 | 0.915 |
| Total plate count | 10.251 | 0.811 | 7.905 | -201.417 | 0.717 | 0.230 | 4.840 | 0.805 |
| Yeast and mould | 11.689 | 0.991 | 9.343 | -200.817 | 0.986 | 0.201 | 5.507 | 0.992 |

Note: R^2^, coefficient of determination; ΔH, enthalpy of activation; △S, entropy of activation; Z, storage temperature span necessary for a tenfold decrease in the D value; D_ref_, D value at the reference temperature.

**SI Table 5.** The optimized microgreen-based dairy (lassi) beverage cost estimation

| **Ingredients** | **Amount/ 1 kg** | **Cost (**₹**)** |
| --- | --- | --- |
| Microgreens (Radish sango) | 92.00 g | 42.00 |
| Dahi | 600.00 g | 45.00 |
| CMC | 2.50 g | 8.47 |
| Sugar | 150.00 g | 12.75 |
| Water | 153.11 ml | 1.53 |
| Packaging cost | | 15.00 |
| Processing cost | | 124.75 |
| Marketing and distribution expenses at 25% of the product | | 124.75+31.19=155.94 |
| The profit margin at 30% of cost of product | | 155.94+46.78=202.72 |
| Total | | 202.72 |
| Production cost for 200 ml | | 40.54 |
